# Cross-Center Vision–Language Transformer for Robust Mammography-Based Breast Cancer Diagnosis

**DOI:** 10.3390/bioengineering13060653

**Published:** 2026-05-31

**Authors:** Anas W. Abulfaraj

**Affiliations:** Department of Information Systems, King Abdulaziz University, P.O. Box 344, Rabigh 21911, Saudi Arabia; awabulfaraj@kau.edu.sa

**Keywords:** breast cancer diagnosis, mammography, vision–language transformer, cross-center generalization, multimodal learning, probability calibration, clinical decision support

## Abstract

While promising results have been demonstrated for deep learning-based breast cancer diagnosis using mammography, problems persist in approaches that rely primarily on visual information. These problems include inadequate performance across diverse clinical centers, various imaging protocols, scanner types, and patient distributions. Here, we introduce Cross-Center Vision–Language Transformer (CC-VLT), a framework that integrates mammograms and clinical text to enable more robust, guided diagnosis. The framework incorporates a vision transformer for mammograms, a text transformer for salient clinical descriptors, bi-directional cross-modal attention for semantics, and a cross-center feature regularization approach to address the challenge of inter-institutional domain shifts. The framework is tested on a leave-one-center-out basis across several public mammography datasets and significantly outperforms strong baseline models in both intra- and cross-center evaluations. Our framework achieved an accuracy of 90.7% with an intra-center ROC–AUC of 0.951 and cross-center ROC–AUC results of 0.912, 0.927, and 0.934 on the CBIS-DDSM, INbreast, and VinDr-Mammo datasets, respectively. Reliability of the malignancy probability predictions improved, as evidenced by a diminished Expected Calibration Error and Brier Score. Our framework, by designing an effective integrated vision–language interaction model and implementing a cross-center feature regularization approach, sets a benchmark for robust breast cancer diagnosis across diverse clinical environments.

## 1. Introduction

Breast cancer continues to rank as one of the most prevalent causes of death for women who have a confirmed diagnosis of a malignant tumor caused by breast cancer, and therefore, the early detection from national screening program is one of the primary ways in which early diagnosis of breast cancer can improve survival rates and decrease the burden of treatment [1,2]. The most common type of medical imaging used to screen for breast cancer is mammography because of its low cost and ability to identify early detection [3]. However, interpreting mammograms poses a major challenge for physicians because malignancies do not always have distinct characteristics and can appear similar to, or even indistinguishable from, normal structures or benign conditions. The variability in the ability of different radiologists to identify malignancy on mammograms, as well as the increasing demand for breast cancer screenings, presents an opportunity for developing reliable automated computer-assisted diagnostic technology to assist healthcare providers in making appropriate clinical decisions based on a diagnosis of breast cancer [4].

In recent years, deep learning has emerged as a dominant paradigm for automated mammography analysis, demonstrating substantial improvements over traditional handcrafted feature-based methods [5]. Convolutional Neural Networks (CNNs), in particular, have shown strong performance in breast cancer detection and classification tasks by learning hierarchical representations directly from image data [6]. Large-scale studies have reported that CNN-based systems achieve performance comparable to, or in some cases exceeding, that of expert radiologists in controlled evaluation settings [7]. Nevertheless, many of these models are trained and evaluated on single datasets or homogeneous data sources, limiting their ability to generalize across institutions with different scanners, acquisition protocols, and patient populations.

Transformer-based models have been introduced to address limitations of purely convolutional approaches in medical image analysis. Vision Transformers (ViTs) and their hierarchical variants model global context through self-attention. They have shown promise in high-resolution tasks like mammography [8]. Hybrid CNN–Transformer models combine local inductive biases with long-range dependency modeling. These hybrids offer improved stability and performance, especially on limited medical datasets [9]. Despite such advances, most transformer-based mammography models focus only on images. They do not explicitly incorporate the non-visual clinical information that radiologists routinely use.

In clinical practice, mammography interpretation is inherently multimodal. Radiologists rely not only on visual cues but also on structured and unstructured clinical information, such as BI-RADS assessments, lesion descriptors, patient history, and diagnostic impressions. Vision–language models have recently attracted attention across general computer vision and medical imaging by enabling joint reasoning across visual and textual modalities [10,11]. Early applications in medical domains suggest that incorporating language information can improve both diagnostic accuracy and interpretability [12]. However, the integration of vision–language learning into mammography-based breast cancer diagnosis remains largely unexplored.

Another critical challenge in deploying deep learning systems for mammography is cross-center generalization. Models trained on data from a limited number of institutions often degrade in performance when applied to unseen centers due to domain shifts caused by differences in hardware, imaging protocols, and demographic factors [13,14]. While domain adaptation and generalization techniques have been proposed to mitigate such effects, many require access to target-domain data or rely on adversarial training strategies that can be unstable in practice [15]. Moreover, robustness is often evaluated implicitly rather than through principled cross-center validation protocols. Beyond accuracy, the reliability of predicted probabilities is a key requirement for clinical decision support. Poorly calibrated models may produce overconfident predictions, leading to inappropriate clinical actions and reduced trust among practitioners [16,17]. In breast cancer screening, well-calibrated estimates of malignancy probability are essential for risk stratification, triage, and referral decisions. Despite this importance, calibration behavior is rarely analyzed in depth in existing mammography studies.

Most CLIP-style vision–language frameworks focus on global image-text contrastive alignment. In this comparison, the CC-VLT framework uses semantically bidirectional interaction, with focused alignment of mammogram image patches to diagnostic descriptors. This allows for modeling relationships between mammographic structures and definitions of clinical concepts used to assess malignancy. In traditional fusion paradigms, features are combined with either front-end or back-end fusion. Our model integrates alignment of multi-domain semantics and learning of center-invariant representations within a single framework. In contrast to domain-generalization models, where the absence of center-target samples in the training set requires domain adaptation or dimensionality reduction, our model uses cross-modal feature regularization to perform stable first- and second-order alignment of center-invariant features, multi-modally and without the constraint of a target domain center. Our CC-VLT framework is a specialized, multi-modal diagnostic framework for mammograms that enables robust cross-center estimation, controlled interpretability, and the assessment of risk for violations of clinical provisions with varying degrees of acquisition.

This study aims to construct a multimodal vision–language framework for breast cancer diagnosis that facilitates the interpretation of complex, multi-center mammographic datasets [18]. The CC-VLT framework is anticipated to enhance the reliability of diagnosis by combining visual representations captured from mammographic imaging with clinically informative semantic descriptors via bidirectional cross-modal attention and center-invariant feature regularization. Unlike typical mammographic models that focus solely on images, this solution is tailored to address cross-center variability, diverse annotations, and multimodal semantic alignment, which are prevalent in most clinical settings. The study also hopes to achieve greater predictive calibration and cross-center generalization, along with enhanced interpretability of diverse modalities for trustworthy computer-aided diagnosis of breast cancer.

The main contributions of this work are summarized as follows. First, a novel Cross-Center Vision–Language Transformer (CC-VLT) framework is proposed for mammography-based breast cancer diagnosis, explicitly integrating mammography images with associated clinical textual information to achieve semantically grounded, clinically informed predictions. Second, a bidirectional vision–language fusion mechanism is introduced to enable fine-grained alignment between visual mammography patterns and diagnostic language cues, going beyond conventional image-only and naive multimodal fusion strategies. Third, a cross-center feature regularization strategy is developed to address inter-institutional domain shifts, allowing the proposed framework to learn center-invariant representations that generalize effectively to unseen imaging sources. Fourth, a comprehensive experimental evaluation is conducted on multiple public mammography datasets using rigorous leave-one-center-out protocols, demonstrating superior diagnostic performance, robustness, and probability calibration compared to strong baseline methods. Finally, extensive ablation and calibration analyses are provided to validate the contribution of individual components and to highlight the clinical reliability of the proposed framework for real-world decision support.

Vision–language frameworks for medical diagnostics have laid the groundwork for leveraging cross-modal alignment in predictive diagnostic modeling. The CC-VLT represents a novel advancement among these frameworks through three main differentiating concepts. First, contrastive image-text alignment used in most CLIP-based frameworks is performed at the global level. CC-VLT focuses on the bidirectional attention at the patch and token levels of the mammographic image and the diagnostic descriptions. Second, the report-aligned radiology transformer frameworks assume the presence of entire radiology reports. The current framework is not constrained in this way and is developed for heterogeneous mammography datasets that combine structured descriptors, BI-RADS datasheets, and semantic text from templates. Lastly, CC-VLT focuses on integrating cross-modal semantic alignment and cross-center feature regularization. This provides a way for the learned representation to address cross-center acquisition bias, while leaving one center out during the evaluation. The core of this method focuses on descriptor-driven bidirectional attention, while cross-center feature regularization addresses the challenges of robust mammographic diagnostics.

This paper is divided into the following sections: Section 2 gives an overview of the previous work and identifies areas of improvement from current literature, Section 3 outlines the design and approach to learning that will be taken by Cross-Center Vision–Language Transformer, Section 4 discusses findings for all datasets and centers, and Section 5 summarizes and suggests directions for future research.

## 2. Literature Review

Deep learning approaches have been the primary drivers of improvements in breast cancer detection via mammography. These methods have led to greater accuracy by improving visual representations. Historically, early computer-aided diagnostic (CAD) systems [19,20] used conventional (handcrafted) descriptors and classifiers, whereas the predominant methodology today is CNNs, with some new methods using transformer architectures. The period from 2022 to the present has seen many high-capacity models published in the literature, based on extensive public datasets (CBIS-DDSM, INbreast, VinDr-Mammo), achieving very good classification results. Nevertheless, even with all the advancements in ML, many methods remain limited by dataset-dependent assumptions and by their poor handling of inter-center variation, making them unsuitable for day-to-day clinical practice.

Conventional CNN architectures remain strong baselines in mammography analysis. Residual networks leverage identity skip connections to stabilize deep optimization and have demonstrated consistent performance on digitized film and full-field digital mammograms when pretrained on large-scale datasets [21,22]. DenseNet variants further enhance feature reuse through dense connectivity, improving sensitivity to fine-grained lesion patterns such as speculations and micro-calcification clusters [12]. EfficientNet and EfficientNetV2 families introduce compound scaling strategies that balance depth, width, and resolution, achieving competitive results under constrained computational budgets [23]. More recently, ConvNeXt has emerged as a modernized convolutional architecture that integrates transformer-inspired design principles and demonstrates improved robustness on high-resolution medical images, including mammography [24].

Beyond generic CNN backbones, several mammography-specific architectures have been proposed. Attention-based CNNs aim to emphasize diagnostically relevant regions while suppressing background tissue. The Multi-Feature Attention Network (MFAN) integrates channel-wise and spatial attention to enhance lesion discrimination, and reports improved performance on CBIS-DDSM and INbreast [25]. StethoNet employs an ensemble-based CNN framework with view-aware aggregation strategies, achieving strong results on VinDr-Mammo and INbreast [26]. Mammo-Clustering introduces a weakly supervised multi-view context clustering strategy that jointly addresses localization and classification, demonstrating competitive performance on CBIS-DDSM and VinDr-Mammo [27]. MamT^4^ explicitly models dependencies across the four standard mammography views using attention mechanisms, highlighting the importance of cross-view anatomical correspondence in VinDr-Mammo [28,29].

Recently, there has been significant interest in multimodal medical imaging systems due to their ability to combine multiple modalities and integrate them with both visual and textual information. An example of this is BiomedCLIP, a model pre-trained on a large-scale biomedical image-text interdisciplinary dataset, which enables it to perform examinations of various biomedical imaging tasks and assess the quality of its representations and the transfer of semantics across imaging tasks. In recent years, studies have explored multimodal foundation models in breast imaging to assess mammographic density and learn diagnostic representations across various acquisition modalities. The results from these studies showcase the potential of cross-modal semantics to enhance discrimination ability and model robustness compared to pure image models, especially in settings where clinical data are diverse and heterogeneous.

Numerous studies have documented notable improvements in large-scale models that integrate vision and language using contrastive learning objectives [10]. Models based on CLIP have evidenced strong performance in zero-shot and transfer learning within several domains of computer vision. Furthermore, biomedical adaptations such as BiomedCLIP and PubMedCLIP, domain-specifically pre-trained on biomedical image–text corpora, have extended CLIP capabilities to medical imaging [30,31]. Recent research in breast imaging has focused on the use of foundation models for analyzing mammographic density and multimodal breast imaging, and has reported promising results in terms of robustness when models are deployed in heterogeneous imaging contexts. However, most existing vision–language foundation models primarily use global contrastive alignment and do not explicitly integrate center-invariant regularization frameworks for the analysis of multi-center mammography.

Recent advances in multimodal medical vision–language modeling have further accelerated the development of clinically oriented cross-modal representation learning frameworks. Large-scale medical VLMs and multimodal foundation models developed during 2024–2025 have demonstrated promising capabilities for radiology report generation, multimodal reasoning, visual question answering, and semantic medical image interpretation. Recent frameworks such as Med-Gemini [32], CXR-LLaVA [33], MammoVLM [34], OmniV-Med [35], and MedMoE [36] have explored multimodal alignment strategies integrating medical imaging with large language models and transformer-based semantic reasoning. These approaches primarily focus on large-scale multimodal foundation modeling, report generation, or generalized multimodal clinical understanding across heterogeneous imaging modalities. In contrast, the proposed CC-VLT framework specifically emphasizes cross-center domain generalization, multimodal semantic alignment for mammography screening, and calibrated probability estimation under heterogeneous acquisition environments. The proposed framework therefore complements recent medical VLM research by targeting robust and clinically reliable multimodal mammography diagnosis under unseen institutional deployment conditions.

Transformer-based and hybrid CNN–Transformer models have also gained traction in recent mammography studies. ViTs and Swin-based variants have been explored to capture long-range contextual dependencies across large mammography fields [37]. Hybrid designs combining convolutional feature extractors with transformer encoders have shown improved stability and performance on limited medical datasets by leveraging both local inductive biases and global context modeling [9]. The use of multiple views with transformers has also shown that looking at how different views are related to one another (the relationship between different views) leads to an improvement in the diagnosis of cancers when they are viewed as screening tests, as some of the lesions may be small or only partially visible [38]. Another common way to improve the ability to differentiate mammography features using automated methods is to combine CNNs with meta-heuristics to optimize feature selection [39]. Table 1 presents the results of an analysis of representative mammography classification methods published between 2022 and the present, detailing their architectural paradigms, assumptions about the modality, and average performance across several major public datasets. As such, the table can be used to identify emerging trends and limitations in the current methodologies researchers use to classify mammograms.

Although there have been advances in research on breast cancer diagnosis, several areas are lacking. First, most methods currently use only the visual aspects of mammograms, although mammography involves not just visualization but also verbal descriptions that accompany mammograms and guide healthcare professionals in making clinical decisions. Second, the majority of methods have used either a single dataset or a dataset combining multiple datasets to develop and evaluate their methods, without addressing how differences in dataset location introduce variability when moving from one center to another. This has resulted in less robustness and effectiveness of these methods when evaluated with a dataset from an unknown center. Third, while multi-view and attention methods improve spatial reasoning and enable a more detailed understanding of mammogram visual features, these approaches do not provide explicit semantic grounding or cross-modal alignment. A final point is that these methods generally evaluate robustness or generalizability implicitly rather than using the standard, established best practices for conducting robust cross-institution validations.

To address the issues described above, the Cross-Center Vision–Language Transformer (CC-VLT) is designed and implemented as a unified framework that leverages an integrated model of mammography images and the descriptive clinical text that describes them. By modeling the two types of data together, the CC-VLT introduces explicit semantic grounding into the clinical decision-making process. Bidirectional vision–language attention enables detailed correspondence between mammogram visual features and the clinical concepts described in the clinical text. The CC-VLT will mitigate the variability introduced by center-related differences and, thus, provide clinicians with robust, interpretable, and generalizable methods/techniques for diagnosing breast cancer, and ultimately be deployed to routine clinical practice within a multi-center environment.

## 3. Proposed Methodology

**Figure 1:**
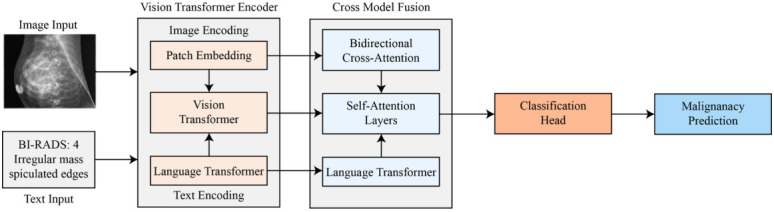
Overview of the proposed CC-VLT framework for mammography-based breast cancer diagnosis.

The CC-VLT framework is designed for domain generalization rather than typical domain adaptation. Let us assume that training source centers make up the set $D_{s}=\left\{D_{1},D_{2},\ldots ,D_{C_{S}}\right\}$, with each center representing a particular sample distribution with scanner characteristics, imaging protocols, and population demographics. The aim of this framework is to develop a generalized prediction function F(·), such that robust diagnostic performance is achievable with a center that is not within the training source centers, i.e., $D_{t}\notin D_{s}$, when an inference is performed. Compared with domain adaptation techniques, the proposed framework does not rely on examples from target domains, target-domain labels, or adversarial alignment during training. The framework implements center-invariant representation learning, multimodal semantic alignment, and cross-center feature regularization, thereby improving resilience against acquisition methods not previously accounted for. An overview of how CC-VLT fits into the mammography analysis pipeline is shown in Figure 1. It shows that CC-VLT uses transformer-based visual feature encoding, multimodal vision–language reasoning, and center-invariant learning strategies to produce stable performance when applied to mammogram analysis across different institutions.

### 3.1. Datasets and Materials

This section describes the datasets and materials used to develop and evaluate the CC-VLT framework for mammography-based breast cancer diagnosis. The experimental design targets multi-center robustness, so datasets were chosen for heterogeneity in imaging protocols, scanner vendors, population demographics, and annotation practices. The experimental corpus is denoted D and is composed of center-specific subsets $D_{c}$, each from a distinct source. All datasets include mammography images and diagnostic labels, while some also provide textual annotations leveraged by the vision–language modeling component:

(1) $$D=\overset{C}{\underset{c=1}{⋃}}D_{c}$$

Each center-specific dataset $D_{c}$ is a collection of triplets $\left(I_{c}^{\left(n\right)},T_{c}^{\left(n\right)},y_{c}^{\left(n\right)}\right)$, where $I_{c}^{\left(n\right)}\in R^{H_{c}\times W_{c}}$ is the *n*-th image from center *c*, $T_{c}^{\left(n\right)}$ is the associated clinical text or metadata if available, and $y_{c}^{\left(n\right)}$ is the ground-truth label. The index *c* is retained during training for calculating center-wise statistics and for cross-center regularization, as outlined in the methodology:

(2) $$D_{c}={\left\{\left(I_{c}^{\left(n\right)},T_{c}^{\left(n\right)},y_{c}^{\left(n\right)}\right)\right\}}_{n=1}^{N_{c}}$$

The primary dataset is the Curated Breast Imaging Subset of the Digital Database for Screening Mammography (CBIS-DDSM) [40], containing digitized film mammograms with pathology-verified benign and malignant labels. Due to differences in older imaging methods, digitization errors, and uneven breast density distribution, CBIS-DDSM is well-suited for assessing a system’s performance against biases in image acquisition. The dataset’s diagnostic category is y∈0,1, with 0 for benign and 1 for malignant tumors:

(3) $$y_{CBIS}\in 0,1$$

To enhance the capabilities of the CBIS-DDSM using modern full-field digital mammograms, we have included the INbreast [41] Dataset as our second center. The INbreast Dataset consists of a collection of high-resolution digital mammograms with associated BI-RADS assessments completed at a European screening center, along with lesion annotations. Compared to the dataset from the CBIS-DDSM Project, the INbreast Dataset has varied intensity distributions, spatial resolutions, and levels of granularity for lesion annotation. The differences in the INbreast Dataset and the CBIS-DDSM Project make it an important opportunity for performing cross-center comparisons. In addition to using the BI-RADS labels for multi-class analysis, we have also mapped them to binary diagnostic categories for consistent labeling across both datasets:

(4) $$Y_{INB}\subseteq BI-RADS1,\ldots ,5$$

The Breast Cancer Digital Repository (BCDR) [42] dataset provides a large collection of mammography images acquired across various clinical environments with different scanners and imaging techniques, along with associated structured diagnostic metadata and pathology-confirmed labels. This added source of heterogeneity (portability), when combined with the multi-center setup used in this research, demonstrated that there were many differences between the BCDR dataset and the two prior datasets of the study (CBIS-DDSM and INbreast):

(5) DBCDR∩DCBIS=⌀

The VinDr-Mammo dataset [43] is provided as a supplementary resource for the vision–language learning paradigm when textual annotations are needed. The VinDr-Mammo dataset contains a wide range of full-field digital mammograms annotated by radiologists from various locations. These annotations used the standard BI-RADS descriptors and report structures. Therefore, the textual descriptors were normalized and mapped to the shared clinical vocabulary V used for language encoding, thereby maintaining consistency in meaning despite differing annotation formats across datasets:

(6) $$T_{c}^{\left(n\right)}\in V^{M_{c}^{\left(n\right)}},\;V=BI-RADSterms,lesiondescriptors,diagnosticcues$$

All mammography images throughout all datasets are unified to the same grayscale format and standardized using the same preprocessing pipeline already outlined, producing standardized, normalized images $I_{c}^{*}\in R^{H_{0}\times W_{0}}$ Textual annotations that are missing from a specific dataset are represented using textual proxies based on templates or labels. By using this design, the proposed CC-VLT architecture can be trained and evaluated in both vision-only and vision + language configurations without introducing any novel external information:

(7) $$I_{c}^{*}=P\left(I_{c}\right),\;\forall c\in 1,\ldots ,C$$

The cumulative effects of the CBIS-DDSM, INbreast, BCDR, and VinDr-Mammo datasets create an experimental setup that yields a wide range of actual variability in mammography images and in how these images are interpreted. Such an extensive and varied collection of data across multiple centers enables a robust evaluation of the proposed cross-center feature regularization and vision–language integration techniques; any increase in performance will be considered legitimate given the methodology employed rather than the source data.

Datasets in this study do not include completed free-text radiology reports. However, they have some structured diagnostic descriptors. These descriptors include Bi-RADS, lesion shape, mass margins, calcification, and pathology-related descriptors. In datasets that do not include these reports, free-text radiology reports are simulated. These reports are created using templates customized to the data. These templates incorporate the metadata that prevails in the data as well as the diagnostic descriptors. The templates are designed to preserve the centers’ vocabulary, helping maintain the diagnostic and mammographic findings. Therefore, the framework should be considered a semantically directed multimodal learning framework. The framework is not designed to analyze free-text radiology reports. Unlike other methods that rely on simple concatenation of diagnostic descriptors, transformer-based language encoders are designed to model interactions among diagnostic descriptors and, therefore, enable alignment across modalities related to mammographic findings.

Table 2 illustrates the differences in the amount of textual dataset information. CBIS-DDSM dataset captures pathology and lesion-related metadata, but lacks free-text radiology reports. In contrast, the INbreast dataset provides some BI-RADS descriptors. Among these three datasets, VinDr-Mammo captures the most textual information, providing the assessment descriptors, findings, and reports developed by radiologists. To facilitate harmonized multimodal learning across datasets, we developed semantic templates for datasets lacking textual radiology reports. These templates were built based on existing dataset data and did not incorporate external clinical data.

Across the entire experimental corpus, around 68% of the samples incorporated structured, template-based, metadata-derived semantic descriptors, while around 32% consisted of partially or fully human-derived radiological descriptors from dataset-provided annotations. The highest proportion of genuine text data was observed in VinDr-Mammo, while the lowest was observed in CBIS-DDSM. The figures pinpoint weaknesses in existing publicly available mammography datasets regarding the availability of reports at scale and encourage the development of innovative multimodal benchmarks with corresponding imaging and report data.

The proposed framework prioritizes adequate discrimination between benign and malignant lesions under different assessment conditions rather than fine-grained classification. More suspicious and malignant findings were grouped into the malignant class, and findings were grouped into the benign class. Bridging a gap in the literature, the proposed binary classification balances the clinical significance of the BI-RADS categories while remaining within the assessment role and focus. The framework also facilitates assessment and provides a free tool to help in the evaluation process, whilst rushing from the clinical evaluation to the assessment of findings. To advance beyond this preliminary assessment, future studies may extend the framework by using ordinal or multi-class BI-RADS predictions to reduce the gap between the diagnostic order and the radiologist’s assessment progression.

Demographic and clinical metadata were included in the analysis when they were annotated in the original dataset. These attributes, which included patient age, breast density descriptors, lesion characteristics, and BI-RADS assessments, were not treated as independent tabular predictors. Instead, they were integrated into the clinical semantic framework and language encoder. Designing the framework in this manner enabled clinical contextual metadata and demographic data to support multimodal semantic reasoning, while also enabling the use of a vision–language integrated framework. Due to variations in the completeness and availability of demographic metadata across participating datasets, these attributes were considered optional and not required for all datasets.

Due to the varied styles of annotations, descriptors, and reporting conventions across the participating mammography datasets, a standardized clinical vocabulary normalization approach was adopted prior to language tokenization and multimodal fusion. In this context, BI-RADS descriptors, lesion morphology, calcifications, and diagnostic annotations, which differ in verbal formulation across centers, were consolidated into a unified terminology space. Here, for example, variant descriptors were grouped into a single category to reflect the clinical finding within a semantic diagnostic framework. As part of this process, the normalization also aligned differences in the case of letters, abbreviations, punctuation, and formatting of descriptors across the datasets. The construction of this harmonized vocabulary, coupled with language embedding, mitigated the misalignment across modalities in diverse annotations tailored to multi-center mammography datasets.

### 3.2. Mammography Image Preprocessing

Preprocessing of mammogram images in the proposed approach is an important preliminary step that affects both the strength and generalizability of the ensuing vision–language learning components across centers. Each mammogram is represented as $I_{c}$ in the dimensional representation $R^{H\times W}$, where *H* and *W* correspond with the height and width originally measured when the image was captured from center c∈1,2,…,C, and the intensity of each pixel is defined as grayscale and continuous. Since there are varying means of acquiring mammograms across centers, there can be large variations in the type of scanner used to obtain the image, as well as in the processes used to calibrate the scanner and overlay the annotations. The resulting image from all of these factors is $I_{c}$, and it often contains large areas of non-breast tissue or background; as well as artifacts from the imaging process—both of which create false correlations between the breast tissue and associated features, therefore confusing downstream processes of model development. To resolve this, an intensity-based masking process is first applied to $I_{c}$ which separates the region of breast tissue from the rest of the background by thresholding on low-intensity background pixels, while keeping all of the anatomically meaningful structures in the breast region, resulting in the image $I_{c}^{m}$. The application of intensity-based masking limits the amount of structural variability while preserving diagnostically relevant features such as masses, calcifications, and architectural distortions:

(8) $$I_{c}^{m}\left(x,y\right)=I_{c}\left(x,y\right)\cdot ⊮\left(I_{c}\left(x,y\right)>\tau_{c}\right)$$

Let (x,y) denote a pixel’s coordinates. The term ⊮(·) represents an indicator function. The term $\tau_{c}$ refers to a center-adaptive intensity threshold that is derived from the histogram distribution of image $I_{c}$. The threshold $\tau_{c}$, specific to center *c*, was derived from the grayscale distribution of mammograms belonging to center *c*. From the image histogram, the background was identified as a low grayscale mode. To separate the breast tissue from the scanner background, $\tau_{c}$ was determined based on a fixed lower-percentile intensity according to center c. Because mammography datasets vary with scanner calibration, digitization, and contrast, a percentile-based approach was used rather than a single global threshold. After thresholding, regions without connectivity were removed, and continuous breast-region masks were created using morphological closing. The thresholding step was used for background suppression, and diagnostic labels and lesion annotations were not utilized in this step. After artifact suppression, image intensity normalization across scanners will be performed to reduce scanner-dependent intensity variations and enhance tissue visibility. The image $I_{c}^{m}$ is normalized for center-wise intensity and converted into a contrast-standardized image $I_{c}^{n}$ so that the dynamic range of all images is the same for each center:

(9) $$I_{c}^{n}=\frac{I_{c}^{m}-\mu_{c}}{\sigma_{c}}$$

The terms $\mu_{c}$ and $\sigma_{c}$ are used in this form to specify the mean and standard deviation of the pixel intensities for all pixels located in the area of the breast centered at the given point *c*, respectively. The de-normalized image $I_{c}^{n}$ is then resized to a fixed spatial resolution of $\left(H_{0},W_{0}\right)$ with bilinear interpolation to be able to fit the vision transformer patch embedding mechanism. To mitigate the risk of information leakage during cross-center evaluation, normalization statistics $\mu_{c}$ and $\sigma_{c}$ were derived solely from the training partition mammography images in each leave-one-center-out fold. Neither validation nor test samples from the respective target centers were used to estimate the preprocessing parameters. The derived normalization statistics were then applied to the relevant validation and testing sets of the same experimental fold. This normalization strategy, which accounts for sample partitioning, prevents the unintentional inclusion of target-domain information during preprocessing. Furthermore, it facilitates the evaluation of cross-center generalization performance in a methodologically sound manner. Let R(·) be the resizing operator, then the resized image $I_{c}^{r}=R\left(I_{c}^{n}\right)$, so that all of the images in the dataset have the same spatial dimensions:

(10) $$I_{c}^{r}=R\left(I_{c}^{n}\right),\;I_{c}^{r}\in R^{H_{0}\times W_{0}}$$

Our approach is to add stochastic data augmentation during training to improve the algorithms’ generalization and reduce overfitting caused by center-specific acquisition patterns. The different types of augmentation we apply include minor affine transformations, intensity changes, and horizontal mirroring. All augmentations we apply are anatomically plausible and combine to form an overall model that is not biased toward the selected augmentation methods. We denote the augmentations we apply by $A(\cdot ;\theta_{a})$, where $\theta_{a}$ is a random variable sampled from a predetermined distribution. The final preprocessed image $I_{c}^{*}$ used for model training is thus defined as

(11) $$I_{c}^{*}=A\left(I_{c}^{r};\theta_{a}\right)$$

**Figure 2:**
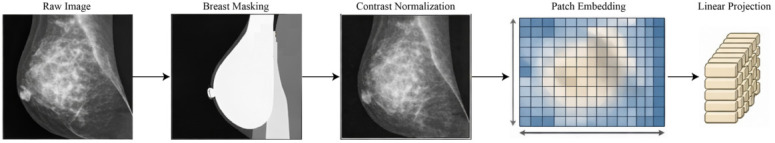
Mammography preprocessing and patch embedding pipeline used before transformer-based visual encoding. Standardized preprocessing reduces center-dependent variability, while fixed-size patch embedding enables token-based learning.

This preprocessing pipeline ensures that all mammography images are transformed into a standardized, center-invariant representation while retaining clinically relevant visual cues. The resulting images $I_{c}^{*}$ form the input to the visual feature extraction module described in the next subsection, thereby establishing a consistent and mathematically well-defined interface between preprocessing and downstream representation learning components. The complete preprocessing and patch embedding pipeline is illustrated in Figure 2.

While fine lesions bordering microcalcifications are highly sensitive to resampling, bilinear interpolation was used in this case, with the understanding that it is a structurally conservative, stable, and compatible operation for transformers and their embedded patch requirements. The preprocessing module deliberately retains high spatial resolution to minimize distortion of structures critical to diagnosis. This also allows for processing multiple heterogeneous datasets while maintaining the required input dimension. Furthermore, the primary purpose of the proposed framework is to achieve effective and consistent cross-center representation learning, both locally and globally, rather than improving pixel-level microcalcifications detail. Compared to nearest neighbors, bilinear interpolation offers more stable optimization during large-scale, multi-modal transformer training, providing smoother transitions with less aliasing.

### 3.3. Visual Feature Extraction

Visual feature extraction in the proposed methodology is performed using a vision transformer architecture specifically tailored to model high-resolution mammography structures while maintaining compatibility with the standardized preprocessing pipeline described in the previous subsection. Let the preprocessed mammography image be denoted as $I_{c}^{*}\in R^{H_{0}\times W_{0}}$, where $H_{0}$ and $W_{0}$ represent the fixed spatial resolution enforced during preprocessing. The image is first decomposed into a sequence of non-overlapping patches of size P×P, yielding a total of $N=\frac{H_{0}W_{0}}{P^{2}}$ patches. According to the suggested implementation, mammography images were split into non-overlapping patches of size P=16×16 pixels prior to the transformer embedding. The patch size is a compromise between preserving local structures relevant to diagnosis and practicality, given the high-resolution analysis of mammography images. Each patch is flattened into a vector $x_{i}\in R^{P^{2}}$, where i∈1,2,…,N indexes the spatial location of the patch within the image. This patch-based decomposition enables the model to capture localized tissue characteristics while remaining computationally tractable for transformer-based processing:

(12) $$x_{i}=Flatten\left(I_{c}^{*}\left[\left(i-1\right)P:iP,,\left(j-1\right)P:jP\right]\right)$$

Each flattened patch vector $x_{i}$ is then linearly projected into a *D*-dimensional latent embedding space using a learnable projection matrix $W_{p}\in R^{D\times P^{2}}$ and bias term $b_{p}\in R^{D}$. This operation transforms raw pixel intensities into semantically meaningful representations suitable for self-attention modeling. The resulting patch embeddings are denoted $z_{i}^{\left(0\right)}\in R^{D}$ and form the initial input sequence to the vision transformer encoder:

(13) $$z_{i}^{\left(0\right)}=W_{p}x_{i}+b_{p}$$

To preserve spatial information that would otherwise be lost due to the permutation-invariant nature of self-attention, learnable positional encoding is incorporated into the patch embeddings. Let $e_{i}\in R^{D}$ denote the positional embedding corresponding to the *i*-th patch location. The position-aware patch representation ${\tilde{z}}_{i}^{\left(0\right)}$ is obtained by element-wise addition of the positional encoding and the projected patch embedding:

(14) $${\tilde{z}}_{i}^{\left(0\right)}=z_{i}^{\left(0\right)}+e_{i}$$

The sequence $\tilde{z}i^{\left(0\right)}{i=1}^{N}$ is then processed by a stack of *L* transformer encoder layers, each composed of a multi-head self-attention (MHSA) module followed by a position-wise feed-forward network. Within the *ℓ*-th encoder layer, the self-attention mechanism computes contextualized representations by modeling pairwise interactions between all patch embeddings. For each attention head h∈1,2,…,H, query, key, and value vectors are generated using learnable projection matrices $W_{Q}^{\left(h\right)}$, $W_{K}^{\left(h\right)}$, and $W_{V}^{\left(h\right)}\in R^{D_{h}\times D}$, where $D_{h}=D/H$:

(15) $$Q_{i}^{\left(h\right)}=W_{Q}^{\left(h\right)}{\tilde{z}}_{i}^{\left(\ell -1\right)},\;K_{i}^{\left(h\right)}=W_{K}^{\left(h\right)}{\tilde{z}}_{i}^{\left(\ell -1\right)},\;V_{i}^{\left(h\right)}=W_{V}^{\left(h\right)}{\tilde{z}}_{i}^{\left(\ell -1\right)}$$

The attention weights between patches *i* and *j* are computed using scaled dot-product attention, allowing for the model to emphasize diagnostically relevant spatial regions while suppressing background noise. The output of each attention head is then obtained as a weighted sum of value vectors across all patches:

(16) $$\alpha_{ij}^{\left(h\right)}=\frac{\exp\left(\frac{Q_{i}^{\left(h\right)}\cdot Kj^{\left(h\right)}}{\sqrt{D_{h}}}\right)}{\sum {k=1}^{N}\exp\left(\frac{Q_{i}^{\left(h\right)}\cdot K_{k}^{\left(h\right)}}{\sqrt{D_{h}}}\right)}$$

(17) $$oi^{\left(h\right)}=\sum {j=1}^{N}\alpha_{ij}^{\left(h\right)}V_{j}^{\left(h\right)}$$

The outputs of all attention heads are concatenated and linearly transformed to form the final output of the multi-head self-attention module. Residual connections and layer normalization are applied to stabilize training and facilitate gradient flow through deep transformer stacks:

(18) $$z_{i}^{\left(\ell \right)}=LN\left({\tilde{z}}_{i}^{\left(\ell -1\right)}+W_{o}\left[o_{i}^{\left(1\right)}\left|\ldots \right|o_{i}^{\left(H\right)}\right]\right)$$

**Figure 3:**
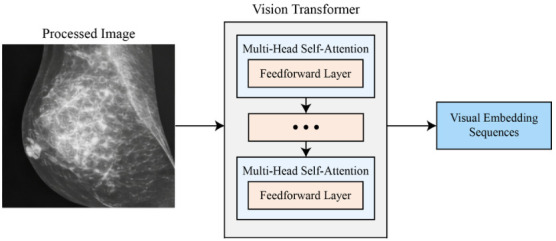
Vision transformer architecture for mammography feature extraction. Multi-head self-attention layers capture both local lesion characteristics and global anatomical context.

After *L* encoder layers, the final visual feature representation is obtained as $Z_{c}=zi^{\left(L\right)}{i=1}^{N}$, where each embedding encodes both local lesion-level characteristics and global anatomical context. These visual embeddings serve as the foundation for the subsequent cross-modal vision–language fusion module, enabling explicit alignment between mammography visual evidence and clinical semantic information. As depicted in Figure 3, the vision transformer models long-range dependencies via multi-head self-attention.

### 3.4. Clinical Language Feature Encoding

The proposed methodology’s clinical language feature encoding is designed to capture rich semantic and contextual information embedded in radiology reports and diagnostic annotations associated with mammography examinations, thereby complementing the visual representations extracted in the previous subsection. Let the clinical text corresponding to an exam acquired from center *c* be denoted as a token sequence $T_{c}=w_{1},w_{2},\ldots ,w_{M}$, where *M* represents the number of tokens obtained after preprocessing operations such as lower-casing, punctuation removal, and sub-word tokenization. Each token $w_{i}$ belongs to a predefined vocabulary V and encodes clinically meaningful concepts, including BI-RADS categories, lesion descriptors, and diagnostic impressions. The objective of this encoding stage is to transform the discrete token sequence $T_{c}$ into a continuous semantic representation that can be explicitly aligned with the visual embeddings $Z_{c}$ defined in the visual feature extraction module:

(19) $$T_{c}=w_{i}|w_{i}\in V,;i=1,\ldots ,M$$

A dense embedding vector $t_{i}\in R^{D_{t}}$ is generated for every token $w_{i}$ by a learnable embedding matrix, $E\in R^{\left|V\right|\times D_{t}}$ where $D_{t}$ represents the dimensionality of the embedding space for languages. The combination of these usable embeddings creates an embedding that preserves and conveys the various syntactic and semantic characteristics of clinical vocabularies and enables gradient-based optimization. Positional embeddings $p_{i}\in R^{D_{t}}$ which maintain the sequential placement of the tokens will then be added on top of this embedding, creating ${\tilde{t}}_{i}^{\left(0\right)}$ as a position-aware token embedding representation produced by adding both positional and dense embeddings to each token:

(20) $${\tilde{t}}_{i}^{\left(0\right)}=E\left(w_{i}\right)+p_{i}$$

The series of vectors from the sequence $\tilde{t}i^{\left(0\right)}{i=1}^{M}$ is then entered into a series of transformer encoder layers $L_{t}$, consisting of the multi-head self-attention mechanism and the feed-forward network. As in the visual transformer architecture, the self-attention mechanism enables the language transformer to learn relationships among clinical terms over long distances, such as between a lesion diagnostic feature and a malignancy assessment. For each language transformer layer *ℓ*, for each layer’s attention head *h*, the attention mechanism computes query, key, and value vectors using a vector attention projection matrix $W{Q,t}^{\left(h\right)}$, $W{K,t}^{\left(h\right)}$,$W{V,t}^{\left(h\right)}\in R^{Dt,h\times D_{t}}$, where $D_{t,h}=D_{t}/H_{t}$ and where $H_{t}$ is the number of attention heads:

(21) $$Q{i,t}^{\left(h\right)}=W{Q,t}^{\left(h\right)}\tilde{t}i^{\left(\ell -1\right)},\;K{i,t}^{\left(h\right)}=W{K,t}^{\left(h\right)}\tilde{t}i^{\left(\ell -1\right)},\;V{i,t}^{\left(h\right)}=W{V,t}^{\left(h\right)}{\tilde{t}}_{i}^{\left(\ell -1\right)}$$

The scaled dot-product attention method provides a way to calculate similarity between 2 tokens. The calculated attention coefficients for tokens *i* and *j* can be used by the model to enhance clinically relevant interactions between the two tokens and reduce irrelevant language. This is especially important for accurately interpreting sentences in medical documents, as slight differences in wording can drastically alter the way a physician interprets diagnostic results:

(22) $$\beta_{ij}^{\left(h\right)}=\frac{\exp\left(\frac{Q{i,t}^{\left(h\right)}\cdot K{j,t}^{\left(h\right)}}{\sqrt{D_{t,h}}}\right)}{\sum_{k=1}^{M}\exp\left(\frac{Q{i,t}^{\left(h\right)}\cdot K{k,t}^{\left(h\right)}}{\sqrt{D_{t,h}}}\right)}$$

(23) $$o{i,t}^{\left(h\right)}=\sum {j=1}^{M}\beta_{ij}^{\left(h\right)}V_{j,t}^{\left(h\right)}$$

The outputs from all attention heads are concatenated and linearly transformed to obtain the contextualized token representation for the *ℓ*-th layer. Residual connections and layer normalization are applied to stabilize training and ensure efficient information propagation across deep language transformer stacks:

(24) $$ti^{\left(\ell \right)}=LN\left(\tilde{t}i^{\left(\ell -1\right)}+Wo,t\left[o{i,t}^{\left(1\right)}\left|\ldots \right|o_{i,t}^{\left(H_{t}\right)}\right]\right)$$

**Figure 4:**
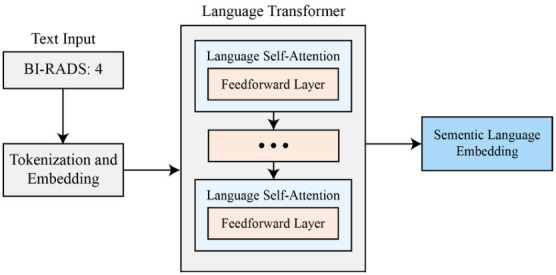
Clinical language feature encoding using a pretrained language transformer. Tokenization and self-attention layers map diagnostic descriptor alignment descriptors into a semantic embedding space suitable for multimodal alignment.

After $L_{t}$ transformer layers, the final language representation is obtained as $T_{c}=ti^{\left(L_{t}\right)}{i=1}^{M}$. These embeddings encode high-level clinical semantics and contextual dependencies, explicitly aligned with the visual embeddings $Z_{c}$ in the subsequent cross-modal fusion module. By integrating structured clinical knowledge with mammography visual evidence, the proposed language-encoding stage provides a semantically rich foundation for clinically meaningful and interpretable breast cancer diagnosis. The clinical language encoding pipeline is illustrated in Figure 4, enabling semantic representation learning for multimodal reasoning.

BioBERT, a pretrained model, offers a significant competitive advantage for the proposed CC-VLT framework for representing clinical language. BioBERT was chosen because general language translation models do not provide the same semantic context for the medical terms, BI-RADS descriptions, lesion attributes, and diagnostic statements that are prevalent in the narrative of clinical mammogram reports. Additionally, the pre-trained biomedical language representations refine the clinical descriptor tokens and help align mammographic visual patterns within the proposed multimodal fusion framework. The clinical language encoder is constructed using a BioBERT backbone consisting of $L_{t}=12$ transformer encoder layers and $H_{t}=12$ multi-head self-attention heads. This design was chosen to ensure alignment with the pretrained BioBERT architecture and to provide adequate representational capacity to handle context and relationships among BI-RADS descriptors, lesion features, and the diagnostic semantics in clinical comments related to mammography.

### 3.5. Cross-Modal Vision–Language Fusion

The cross-modal vision–language fusion module is the core component of the proposed CC-VLT framework, explicitly designed to align visual embeddings extracted from mammography images with semantic representations derived from clinical text. Let the final visual feature sequence obtained from the vision transformer be denoted as $Z_{c}=zi{i=1}^{N}$, where $z_{i}\in R^{D}$ represents the contextualized embedding of the *i*-th image patch, and let the final language feature sequence be denoted as $T_{c}=tj{j=1}^{M}$, where $t_{j}\in R^{D_{t}}$ corresponds to the contextualized embedding of the *j*-th clinical token. The goal of this fusion phase is to map both modalities into a shared latent space and to learn bi-directional interactions between them; that is, to relate specific regions of mammograms to clinically meaningful textual concepts, ultimately enabling semantically rich visual interpretation:

(25) $$Z_{c}=zi\in R^{D}{i=1}^{N},\;T_{c}=tj\in R^{D_{t}}{j=1}^{M}$$

To enable image–text interaction, features from each modality are linearly transformed into a common embedding space of dimension $D_{f}$, using modality-specific weights $W_{z}\in R^{D_{f}\times D}$ and $W_{t}\in R^{D_{f}\times D_{t}}$. The projected representations ${\hat{z}}_{i}$ and ${\hat{t}}_{j}$ are input to the cross-attention mechanism:

(26) $$\hat{z}i=Wzz_{i},\;\hat{t}j=Wtt_{j}$$

The proposed combined approach appears to use Cross-Attention in both directions (i.e., Visual-to-Language and Language-to-Visual), thereby enabling Mutual Refinement between the two modalities. The Emphasis of Visual to Language Cross-Attention is that the input image features are considered as Query Identities, while the text features are used as the Key and Value for this branch. For the *h*-th attention head, the query, key, and value vectors are computed using learnable projection matrices $W{Q,z}^{\left(h\right)}$, $W{K,t}^{\left(h\right)}$, and $W{V,t}^{\left(h\right)}\in R^{Df,h\times D_{f}}$, where $D_{f,h}=D_{f}/H_{f}$ and $H_{f}$ denotes the number of cross-attention heads:

(27) $$Q{i,z}^{\left(h\right)}=W{Q,z}^{\left(h\right)}\hat{z}i,\;K{j,t}^{\left(h\right)}=W{K,t}^{\left(h\right)}\hat{t}j,\;V{j,t}^{\left(h\right)}=W{V,t}^{\left(h\right)}{\hat{t}}_{j}$$

By quantifying the importance of each clinical word relative to a particular image patch, the attention weights provide a means to link mammographic findings to diagnostic meanings (e.g., guidelines) for malignancy and to descriptive descriptors (BI-RADS):

(28) $$\gamma_{ij}^{\left(h\right)}=\frac{\exp\left(\frac{Q{i,z}^{\left(h\right)}\cdot K{j,t}^{\left(h\right)}}{\sqrt{D_{f,h}}}\right)}{\sum_{k=1}^{M}\exp\left(\frac{Q{i,z}^{\left(h\right)}\cdot K{k,t}^{\left(h\right)}}{\sqrt{D_{f,h}}}\right)}$$

(29) $$o{i,z}^{\left(h\right)}=\sum {j=1}^{M}\gamma_{ij}^{\left(h\right)}V_{j,t}^{\left(h\right)}$$

Symmetrically, in the language-to-visual attention branch, language embeddings serve as queries, while visual embeddings act as keys and values, enabling textual tokens to attend to diagnostically relevant image regions. This bidirectional design ensures that both modalities influence each other during representation learning rather than relying on unidirectional conditioning:

(30) $$Q{j,t}^{\left(h\right)}=W{Q,t}^{\left(h\right)}\hat{t}j,\;K{i,z}^{\left(h\right)}=W{K,z}^{\left(h\right)}\hat{z}i,\;V{i,z}^{\left(h\right)}=W{V,z}^{\left(h\right)}{\hat{z}}_{i}$$

(31) $$\delta_{ji}^{\left(h\right)}=\frac{\exp\left(\frac{Q{j,t}^{\left(h\right)}\cdot K{i,z}^{\left(h\right)}}{\sqrt{D_{f,h}}}\right)}{\sum_{k=1}^{N}\exp\left(\frac{Q{j,t}^{\left(h\right)}\cdot K{k,z}^{\left(h\right)}}{\sqrt{D_{f,h}}}\right)}$$

(32) $$o{j,t}^{\left(h\right)}=\sum {i=1}^{N}\delta_{ji}^{\left(h\right)}V_{i,z}^{\left(h\right)}$$

The outputs from all attention heads are concatenated, then transformed by linear layers, and finally passed through residual connections and layer normalization. This ensures stable optimization. The refined embeddings are then pooled to form a unified cross-modal representation $f_{c}\in R^{D_{f}}$, combining information from both modalities.

(33) $$f_{c}=Pool\left(\left[O_{z}|O_{t}\right]\right)$$

Here, $O_{z}$ and $O_{t}$ denote the concatenated outputs of the visual-to-language and language-to-visual attention branches, respectively, and Pool(·) represents a global average or attention-based pooling operation. The resulting fused representation $f_{c}$ serves as the shared latent descriptor, subsequently used for cross-center regularization and diagnostic prediction, enabling the proposed framework to jointly reason over mammography visual evidence and clinical semantic context in a unified and interpretable manner. For the proposed implementation, we used global average pooling to combine the bidirectionally refined visual and language representations into the unified multimodal descriptor $f_{c}$. We chose global average pooling because it provides stable feature aggregation with low parameter memory and a lower risk of overfitting in heterogeneous multi-center training. Global average pooling, compared to attention-based pooling methods, does not add significant optimization complexity while capturing globally contextualized, semantically relevant information, along with the preceding bidirectional cross-attention pooling.

Traditional multimodal frameworks often rely on direct feature integration or delayed prediction fusion to integrate visual and clinical data. Though convenient, these methods fail to capture, or simply ignore, the detailed relationship between the areas of the mammogram on the image and the diagnostic language descriptors. Direct integration merges clinical and visual data but lacks contextual connections. On the other hand, delayed integration combines the independent predictions of the modalities after separate learning processes for the representations. The proposed bidirectional cross-modal attention mechanism offers the flexibility to integrate, at the token level, the visual patterns of mammograms and meaningful clinical language, both of which aid classification. This mechanism helps the framework capture and maintain multimodal relationships, go beyond simple feature integration, improve alignment, and increase the system’s capacity to withstand variations.

### 3.6. Cross-Center Feature Regularization

Cross-center feature regularization is a fundamental component of the proposed CC-VLT methodology, explicitly designed to mitigate performance degradation arising from inter-center domain shifts in multi-institutional mammography data. Let the fused vision–language representation obtained from the previous subsection be denoted as $f_{c}\in R^{D_{f}}$ for a sample acquired from center c∈1,2,…,C. Due to variations in imaging hardware, acquisition protocols, and population characteristics across centers, the empirical distribution of $f_{c}$ may differ significantly, leading to biased decision boundaries and reduced generalization. The objective of cross-center regularization is therefore to enforce distributional consistency among these representations in the shared latent space, while preserving discriminative information relevant to breast cancer diagnosis:

(34) $$f_{c}=\Phi \left(Z_{c},T_{c}\right),\;f_{c}\in R^{D_{f}}$$

Here, Φ(·) is the cross-modal fusion function parameterized by the vision–language transformer, and $Z_{c}$, $T_{c}$ are the visual and language embeddings previously defined. For each center *c*, the latent feature distribution has mean vector $\mu_{c}\in R^{D_{f}}$ and covariance matrix $\Sigma_{c}\in R^{D_{f}\times D_{f}}$, computed over all samples from that center in a mini-batch or epoch:

(35) $$\mu_{c}=\frac{1}{\left|Sc\right|}\sum n\in S_{c}f_{c}^{\left(n\right)},\;\Sigma_{c}=\frac{1}{\left|Sc\right|}\sum n\in S_{c}\left(f_{c}^{\left(n\right)}-\mu_{c}\right){\left(f_{c}^{\left(n\right)}-\mu_{c}\right)}^{⊤}$$

In this formulation, $?[0mS_{c}$ is the set of samples from center *c*, with $?[0mf_{c}^{\left(n\right)}$ as the *n*-th fused embedding. To learn center-invariant features, we introduce a distribution alignment loss. It penalizes differences between center-specific features and a global distribution. The global mean and covariance are aggregated from all training centers:

(36) $$\mu =\frac{1}{C}\sum_{c=1}^{C}\mu c,\;\Sigma =\frac{1}{C}\sum {c=1}^{C}\Sigma_{c}$$

The center combination regularization loss ($L_{cc}$) is calculated by summing the squared difference between all centers’ feature statistics and the overall statistic of the dataset. The loss function encourages both first- and second-order moment alignment of features from different centers to converge towards a common latent structure while not collapsing on top of each other, and maintains the respective discriminative information of the different centers:

(37) $$Lcc=\sum {c=1}^{C}\left({\left|\mu_{c}-\mu \right|}_{2}^{2}+{\left|\Sigma_{c}-\Sigma \right|}_{F}^{2}\right)$$

In this instance, the Euclidean norm is defined through ${\left|\cdot \right|}_{2}$ and the Frobenius norm through |·|F. The model is engineered to reduce the encoding of acquisition artifacts across centers and to improve the representation of disease-related attributes shared across centers by minimizing the loss defined in Lcc. Proposed cross-center regularization is based on moment-based domain alignment, which involves matching distributions’ first and second moments. In the latent feature space, the mean alignment term centers the moments and shifts. The covariance alignment term addresses disparities in feature dispersion and correlations, caused by variability in scanner protocols, acquisition settings, and populations. First and second order moment matching, while not guaranteeing any equivalence of distributions in the non-Gaussian case, offers a controlled, stable, and differentiable approximation of the center’s discrepancy, which can be improved in conjunction with the primary diagnostic task. This is especially important for the proposed CC-VLT framework, as it omits the instability that typically accompanies adversarial domain training and does not require samples from the target domain for testing. Therefore, the proposed regularization framework offers a practical center-invariance constraint that does not allow for a full probabilistic model for all the potential domain shifts. Furthermore, normalizing the loss by the number of centers stabilizes the optimization and reduces the risk of domination by centers with the largest sample sizes. This was implemented by integrating it into the end-to-end objective of the entire training process:

(38) $$\tilde{L}cc=\frac{1}{C}Lcc$$

By implementing the CC-VLT framework, a diagnostic loss function will be enforced as introduced in the next paragraph. This loss function, along with the cross-center regularization mechanism, promotes robustness to domain shifts while maintaining classification performance. By constructing the CC-VLT Framework in this way, it can produce Center-Invariant/Semantically Grounded Representations that generalize very successfully to new institutions, which are critical for ensuring consistent clinical deployment across multi-center diagnostic and screening practice.

The cross-center feature regularization strategy aligns with alignment approaches like CORAL and moment-matching, as it aims to align first- and second-order feature statistics across disparate imaging centers. Unlike traditional CORAL, which is tailored for shallow feature adaptation, the regularization in the framework operates in the multimodal latent space and is jointly optimized through vision–language interaction and diagnostic supervision. The framework also distinguishes itself from domain-general adversarial methods that use domain discriminators and minimax optimization. While strong domain invariance is a characteristic of adversarial methods, they may result in unstable optimization, increased training complexity, and sensitivity to hyperparameter choices when applied to medical imaging with limited data. The employed moment-based regularization method is a preferable alternative to adversarial methods for representation learning, as it is center-invariant and does not require target-domain data or adversarial mechanisms for training.

The moment-based regularization strategy under consideration does not require that multimodal latent representations be Gaussian. Instead, it uses the alignment of first-order and second-order statistics as an approximation that is both practical and computationally efficient for reducing discrepancies in crossing center features. Since matching means and covariances does not ensure a high-dimensional non-Gaussian feature manifold, and empirical studies in the field of domain generalization have shown that moment alignment improves the consistency of representations across different acquisition conditions, the proposed CC-VLT framework posits that the regularization term acts as a soft distributional constraint. This soft distributional constraint will yield center-invariant representation learning without imposing strict probabilistic assumptions on the latent feature space.

### 3.7. Diagnostic Prediction Module

The diagnostic prediction module constitutes the final stage of the proposed CC-VLT framework and is responsible for translating the fused, center-invariant vision–language representation into clinically meaningful malignancy predictions. Let the unified cross-modal feature vector obtained after cross-center regularization be denoted as $f_{c}\in R^{D_{f}}$ for a mammography exam acquired from center *c*. This representation encodes complementary visual and linguistic information while suppressing center-specific biases, thereby serving as a robust input to the prediction head. The objective of this module is to map $f_{c}$ to a probability distribution over predefined diagnostic categories, such as benign and malignant, in a manner that is both discriminative and well-calibrated for clinical decision support:

(39) $$f_{c}\in R^{D_{f}}$$

The prediction head is implemented as a sequence of fully connected layers that progressively transform the fused embedding into a lower-dimensional decision space. Let $hc^{\left(0\right)}=fc$ denote the input to the prediction module. For the *ℓ*-th fully connected layer, the hidden representation $hc^{\left(\ell \right)}\in R^{D\ell }$ is computed using a linear transformation followed by a nonlinear activation function σ(·), such as the rectified linear unit. The learnable parameters of this layer consist of a weight matrix $W\ell \in R^{D\ell \times D_{\ell -1}}$ and a bias vector $b\ell \in R^{D\ell }$:

(40) $$hc^{\left(\ell \right)}=\sigma \left(W\ell hc^{\left(\ell -1\right)}+b\ell \right)$$

To enhance numerical stability and prevent overfitting, layer normalization is applied after each hidden transformation, ensuring that the feature distributions remain well-conditioned during end-to-end optimization. After $L_{p}$ fully connected layers, the final hidden representation $h_{c}^{\left(L_{p}\right)}$ is projected onto a *K*-dimensional logit space, where *K* denotes the number of diagnostic categories defined by the clinical task:

(41) $$\ell_{c}=W_{o}h_{c}^{\left(L_{p}\right)}+b_{o},\;\ell_{c}\in R^{K}$$

Here, $\ell c={\left[\ell c,1,\ell_{c,2},\ldots ,\ell_{c,K}\right]}^{⊤}$ represents the normalized prediction scores for each diagnostic category. These logits are subsequently transformed into a probability distribution using the softmax function, yielding malignancy probabilities that sum to one and can be directly interpreted in a clinical context:

(42) $$p_{c,k}=\frac{\exp(\ell_{c,k})}{\sum_{j=1}^{K}\exp\left(\ell_{c,j}\right)},\;k=1,\ldots ,K$$

In this formulation, $p_{c,k}$ denotes the predicted probability that the input mammography exam from center *c* belongs to diagnostic category *k*. The predicted class label ${\hat{y}}_{c}$ is obtained by selecting the category with the highest posterior probability, although the full probability vector is retained for downstream clinical interpretation and risk stratification:

(43) $$\hat{y}c=\arg\max k;p_{c,k}$$

The diagnostic prediction module is trained jointly with the visual encoder, language encoder, cross-modal fusion module, and cross-center regularization mechanism using a supervised classification loss. Let $y_{c}\in 1,\ldots ,K$ denote the ground-truth diagnostic label for the input sample. The classification loss $L_{cls}$ is defined as the categorical cross-entropy between the predicted probability distribution and the true label:

(44) $$Lcls=-\sum {k=1}^{K}⊮\left(y_{c}=k\right)\log\left(p_{c,k}\right)$$

This loss encourages accurate malignancy prediction while preserving probabilistic calibration, which is critical for clinical decision-making scenarios such as screening prioritization and biopsy recommendation. When combined with the cross-center regularization loss introduced in the previous subsection, the prediction module enables the proposed CC-VLT framework to achieve robust, interpretable, and clinically reliable mammography-based breast cancer diagnosis across multiple centers.

### 3.8. Optimization Objective

The optimization objective of the proposed CC-VLT framework is formulated to jointly enforce accurate mammography-based breast cancer diagnosis and robust generalization across heterogeneous imaging centers. To this end, a composite loss function is defined that integrates the supervised diagnostic classification loss with the cross-center feature regularization term introduced in the preceding subsection. This unified objective enables end-to-end training of all model components, including the visual encoder, clinical language encoder, cross-modal fusion transformer, cross-center regularization module, and diagnostic prediction head. Let the complete set of trainable parameters of the proposed framework be denoted as Θ, encompassing all projection matrices, attention weights, and classifier parameters defined throughout the methodology:

(45) $$\Theta =\Theta vis,\Theta lang,\Theta fuse,\Theta reg,\Theta_{pred}$$

For a training dataset comprising samples from *C* centers, let $D={⋃}_{c=1}^{C}D_{c}$ denote the full dataset, where $Dc=\left(Ic^{\left(n\right)},Tc^{\left(n\right)},y_{c}^{\left(n\right)}\right){n=1}^{N_{c}}$ represents the set of $N_{c}$ labeled samples from center *c*. For each sample, the forward pass of the network produces a predicted probability distribution $pc^{\left(n\right)}=\left[p{c,1}^{\left(n\right)},\ldots ,p{c,K}^{\left(n\right)}\right]$ over *K* diagnostic categories, as defined in the diagnostic prediction module. The supervised classification loss Lcls is computed as the empirical expectation of the categorical cross-entropy over all training samples:

(46) $$Lcls=-\frac{1}{\sum {c=1}^{C}N_{c}}\sum_{c=1}^{C}\sum_{n=1}^{N_{c}}\sum_{k=1}^{K}⊮\left(y_{c}^{\left(n\right)}=k\right)\log\left(p_{c,k}^{\left(n\right)}\right)$$

While Lcls enforces discriminative learning for malignancy prediction, it does not explicitly account for distributional discrepancies across imaging centers. To address this limitation, the cross-center feature regularization loss ${\tilde{L}}_{cc}$ derived in the previous subsection is incorporated into the optimization objective. This regularization term penalizes deviations between center-specific feature distributions and the global latent distribution, thereby encouraging center-invariant representation learning:

(47) $$\tilde{L}cc=\frac{1}{C}\sum {c=1}^{C}\left({\left|\mu_{c}-\mu \right|}_{2}^{2}+{\left|\Sigma_{c}-\Sigma \right|}_{F}^{2}\right)$$

The final optimization goal of $L_{total}$ is a linear combination of the classification loss and a cross-center regularization loss. A non-negative hyperparameter λ is included to provide control over the trade-off between performance relating to diagnostics and performance relating to cross-center robustness. This hyperparameter can be adapted to the degree of heterogeneity present in the training set’s domain, enabling model customization based on this factor:

(48) $$Ltotal=Lcls+\lambda ,{\tilde{L}}_{cc}$$

The training of the proposed framework optimizes the Θ parameters by minimizing the total loss $L_{total}$ using stochastic gradient-based optimization methods, enabling backpropagation across all network components. This backpropagation jointly trains visual and language embeddings with cross-modal interactions and center-invariant constraints in an end-to-end fashion. Minimizing this composite loss enables balancing predictive accuracy and robustness against inter-center variability, enabling the creation of a reliable, generalizable mammography-based diagnostic system for breast cancer that can be used across multiple clinical institutions.

The hyperparameter λ, which determines the extent of the cross-center regularization term, was empirically selected based on the performance of the cross-center model using the leave-one-center-out experimental method. Multiple candidate choices were tested to achieve an optimal balance of diagnostic discrimination and center-invariant representation learning. A small value of λ reduced distribution alignment and weakened cross-center robustness, whereas a large value of λ constrained the representation space to a degree, resulting in a slight decrease in classification performance. The selected value was the one that helped maintain the classification model’s performance, as measured by changes in the classification score and in the distribution of scores, to achieve a balance of classification accuracy, calibration, and cross-center generalization.

## 4. Experimental Results

This section describes the evaluation of the Cross-Center Vision–Language Transformer (CC-VLT) framework for breast cancer diagnosis via mammography. Evaluation was performed to establish diagnostic accuracy, robustness to inter-center domain shifts, and the contributions of each component of the architectural design. All evaluations were performed on preprocessed, standardized multi-center mammography datasets, trained and tested using standard protocols to enable unbiased, consistent evaluations across all centers.

### 4.1. Experimental Setup and Performance Evaluation

All experiments were completed using a uniform framework across the various supported mammography databases described above. This allows for consistent methodology and enables fair analysis of competing methods by using the same protocol. Evaluating cross-center reliability and reproducibility is done using a leave-one-center-out method, in which data from one center are withheld from testing, while data from the others are used for training and validation. This approach simulates how a trained model would be implemented in a clinical setting, where it is applied to previously untested institutions with different acquisition behaviors. To maintain an identical experimental environment, all models go through the same standard preprocessing procedures, network architecture, and optimization parameters. Therefore, the only variable affecting performance is the difference in architecture and methodology between models. In Table 3 is displayed the distribution of training, validation, and testing samples across the entire dataset under the leave-one-center-out experiment setup.

The goal of this study was to provide a fair and reproducible comparison of each baseline model versus our own method. To do this, all experiments were run in the same training and evaluation environment. Specifically, we used the same parameters for the optimization strategy, learning rate policy, batch size, and number of training iterations, and determined all hyperparameters based on validation set performance. After determining the hyperparameters, they were fixed throughout the experiment. Additionally, all training and model evaluation have been performed using the same hardware and software environment to eliminate potential variability arising from the computational environment. The complete list of hyperparameter settings used for model training, along with details of the hardware and software environments used throughout the experimental study, is summarized in Table 4.

The experimental arrangement ensures that the results obtained in the subsequent performance and ablation comparisons were generated in a methodologically reliable manner using the proposed CC-VLT model, rather than being influenced by other architectural factors that may compromise the evaluation. The diagnostic accuracy of the models developed as part of the project will be evaluated using a broad array of common clinical classification metrics to give a comprehensive evaluation of the diagnostic reliability of breast cancer screening. Overall classification accuracy will be represented using an accuracy value. The model’s ability to identify malignant and benign cases will be quantified using sensitivity and specificity, respectively. The precision and F1-score values will also be included to demonstrate the false-positive-to-false-negative ratio. These two metrics are important for reducing the potential for unnecessary biopsies and missed diagnoses. In addition to these metrics, the area under the receiver operating characteristic (ROC) curve will be used to assess the model’s discriminatory power in a threshold-independent way. For the cross-hub experiments, the metrics will be compiled separately for each hub and averaged across all hubs, allowing for an understanding of how well the model performs within a particular hub and how well it is likely to perform in a heterogeneous setting.

Table 5 presents details of the clinical language encoder and descriptor normalization and a pipeline as used within the CC-VLT framework. BioBERT was chosen as the language encoder because it is optimized for the biomedical and clinical domains. As a result of this optimization, BioBERT provides a better semantic space for representing and understanding mammography descriptors compared to other generalized language models. The WordPiece tokenizer, complemented with a vocabulary of 28,996 tokens, provides a strong framework for addressing standard BI-RADS language and descriptors of rarer lesions through subword tokenization. The language embedding was set to $D_{t}=768$, with a maximum sequence length of M=64 tokens to balance computational efficiency with the preservation of pertinent clinical information. To address variations in annotation formats across datasets and the resulting inconsistencies, all lesion descriptors and BI-RADS categories were placed into a unified clinical vocabulary prior to tokenization. To avoid the introduction of spurious information, missing descriptors were denoted as *unknown*. The structured metadata and diagnostic labels were transformed into short, clinically meaningful template sentences, which were then encoded and embedded into the shared fusion space of dimension $D_{f}=512$ before release.

Table 6 presents the computational cost attributed to the language encoder, cross-modal fusion module, and cross-center regularization component. Building off the image-only CC-VLT, the full multimodal model roughly doubles the parameter count from 91.2 M to 201.3 M, primarily due to the BioBERT language encoder. The module contributes to an approximate 7.0 G increase in computational complexity, measured in FLOPs, and a 14.4 ms increase in average inference time. Even though the multimodal framework adds some computational burden, it remains within a realistic range for offline mammography screening and clinically assisted decision-support applications, where diagnostic accuracy and cross-center reliability outrank the need for ultra-low latency.

To achieve balance and methodological fairness, additional multimodal vision–language models and domain-generalization methods adapted to Mammography were tested. With the same preprocessing interventions, optimizations, and center-out-evaluation methods, they were evaluated under the same fair evaluation framework. Leaving aside comparison methods, the same experimental conditions were applied to all of them. The differences in performance were assumed to be due to the models’ architectures and representation-learning traits, rather than to their implementation or training. To assess experimental stability and minimize sensitivity to arbitrary initialization, all tests were run independently across five random seeds. This was done under the same preprocessing, optimization, and leave-one-center-out evaluation settings. In all tables, the reported performance values correspond to the mean and standard deviation of the independent experiments. The random seeds influenced parameter initialization, order of data shuffling, the patterns of stochastic augmentation, and the dynamics of optimization. This was crucial for assessing the model’s reproducibility and performance consistency.

Due to discrepancies in size and class distribution across mammography datasets, optimization strategies were implemented to address bias toward larger centers and reduce imbalance during training. Center-wise balanced sampling was used during mini-batch construction to give smaller datasets consistent representation during training. Weighted classification optimization was used to address class imbalance between benign and malignant cases across centers. The leave-one-center-out evaluation framework further controls center dominance during testing by assessing the ability to generalize to the left-out target center. This approach strengthens the ability to generalize to larger datasets while enabling stable multimodal representation learning across different datasets.

### 4.2. Diagnostic Performance and Cross-Center Robustness

The performance of the proposed CC-VLT framework for diagnostic purposes has been thoroughly evaluated using clinically useful measures, including accuracy, sensitivity, specificity, F1-score, and ROC-AUC. The analysis compares these metrics with results from multiple public mammography databases; therefore, the assessment provides information about the performance of the different datasets relative to their respective datasets and about the overall strength of the CC-VLT framework across a variety of datasets. Results show that the CC-VLT outperforms using images alone across most measured criteria, particularly in terms of sensitivity and ROC-AUC, thereby reducing the number of missed cancer diagnoses and increasing confidence in the diagnosis.

MFAN was selected as the primary attention-based CNN baseline due to its integration of multi-level feature aggregation along with attention-based mammography representation learning, thus synthesizing many attributes of sophisticated breast imaging attention architectures. Several recently published mammography-specific CNN frameworks share similar design concepts, including spatial–channel hybrid attention, multi-scale attention refinement, and advanced hierarchical feature enhancement for lesion localization and classification. Although study designs differ in implementation, the focus of these methods is invariably on enhancing the quality of diagnostic representation through adapted attention-driven feature weighting. Consequently, MFAN is seen as a representative, computationally efficient attention-based benchmark for assessing the developed multimodal transformer framework in both intra-center and cross-center scenarios.

**Figure 5:**
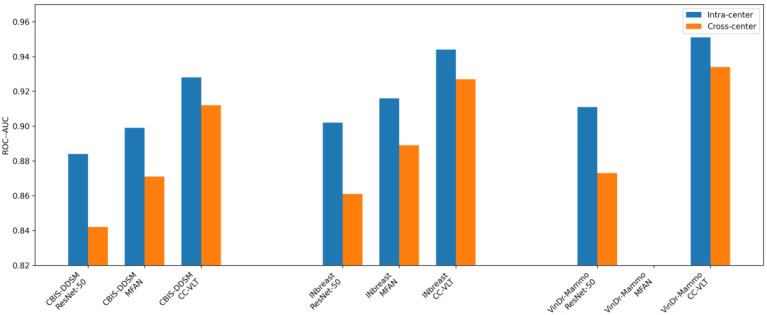
Dataset-wise comparison of ROC–AUC under intra-center and cross-center evaluation settings.

Table 7 summarizes the findings from the intra-center evaluations, in which all models were trained and tested using the same clinical site data sources. The intra-center results were similar across the three datasets and provided a measure of the ability of the different models to harness the specific characteristics of their site-based clinical data without any additional domain-shift considerations. The cc-VLT consistently outperformed all three CNN-based and attention models across all metrics. The performance of all three models still lagged significantly behind that of the CC-VLT model, especially in terms of sensitivity and ROC–AUC scores, two important factors in reducing missed cancer diagnoses. This supports the idea that leveraging vision–language reasoning capabilities gives the CC-VLT framework a more comprehensive understanding of image semantics than those available solely from images, resulting in better diagnostic performance even in the most homogeneous environments. Figure 5 provides a visual comparison of intra-center versus cross-center generalization performance for cc-VLT and other baseline methods across the three datasets CBIS-DDSM, INbreast, and VinDr-Mammo.

Additional ensemble-based, transformer-based, and multi-view mammography architectures were incorporated into the intra-center evaluation to provide a more comprehensive comparison against recent breast imaging frameworks. StethoNet improves diagnostic stability through ensemble aggregation, while MamT4 enhances representation learning by modeling inter-view anatomical correspondence using transformer-based attention. Although these methods outperform conventional CNN baselines, the proposed CC-VLT framework consistently achieves the highest ROC–AUC and sensitivity, indicating that multimodal semantic alignment provides additional diagnostic benefits beyond purely visual ensemble and multi-view modeling approaches.

The results of the Cross Center Evaluation are displayed in Table 8. Each dataset is similarly classified as an “unseen” target domain based on a single-center-out methodology. This difficult approach illustrates how variability introduced into the data by differences in imaging modalities, acquisition methods, and demographics impacts the overall performance of the classifiers included in this study. The drop in the sensitivity coefficient and ROC-AUC confirms that the classification of malignant cases has become less reliable as a result of pooling data from multiple centers. Although MFAN may improve upon image-only results using the attention-based model, it also shows an overall deterioration in performance compared to viewing in a single center. However, the CC-VLT method maintains enhanced performance across multiple sources. The accuracy, sensitivity, F1-score, and ROC-AUC of CC-VLT are substantially better than those of the other methods, demonstrating that CC-VLT leverages cross-center feature regularization and vision–language fusion to produce center-invariant representations. In doing so, CC-VLT has learned how to generalize effectively across very different clinical environments.

**Figure 6:**
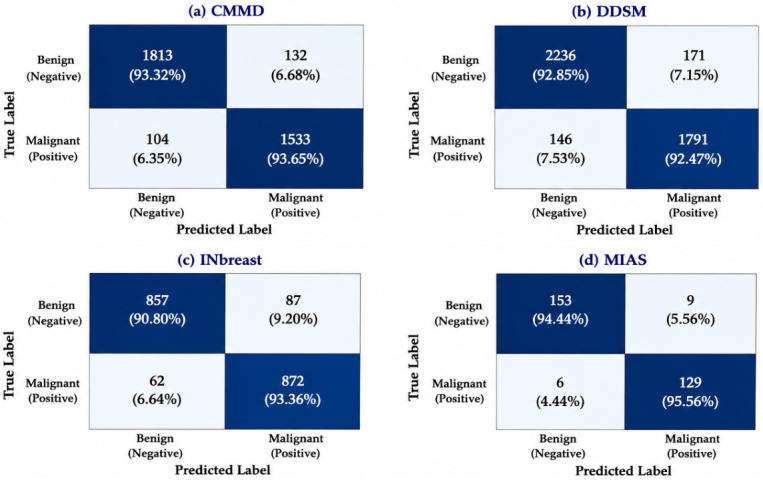
Confusion matrices of the proposed CC-VLT framework across the four mammography datasets: CMMD, DDSM, INbreast, and MIAS.

Under cross-center evaluation, ensemble-based and multi-view transformer architectures demonstrate improved robustness compared with single-backbone CNN models due to their stronger representation stability across heterogeneous imaging environments. Nevertheless, the proposed CC-VLT framework consistently achieves superior cross-center performance across all evaluation metrics. These findings suggest that integrating semantic clinical descriptor alignment with explicit center-invariant feature regularization provides stronger robustness against inter-center domain shifts than ensemble averaging or purely visual transformer-based multi-view modeling alone. Figure 6 shows that the proposed CC-VLT framework demonstrates impressive consistency for malignant and benign classification during both intra-center and cross-center evaluations. The low false-negative rates and controlled false-positive rates demonstrate robust positive malignant lesion detection and consistent diagnostic specificity across variable imaging conditions. The confusion matrices validate the robustness of the proposed multimodal semantic alignment and cross-center regularization strategy across multiple mammography datasets.

### 4.3. Cross-Center Generalization Analysis

In this section, we present an analysis of the diagnostic accuracy of different predictive models using a systematic, center-wise evaluation to determine how well the models can predict the diagnostic outcomes of mammograms held out from the training set by center using a leave-one-center-out evaluation approach. This type of evaluation is more informative than using aggregate metrics across all centers, because it provides more detailed information about the variances in diagnostic performance across individual imaging sources when evaluated as an ’unseen’ target domain. A detailed analysis of center-wise generalization performance is a critical component of understanding the robustness of predictive models for deployment in real-world practice, because acquisition protocols, scanner manufacturers (vendors), and population characteristics vary widely across institutions.

Table 9 summarizes the results of the ROC–AUC analysis for each of the centers using the leave-one-center-out evaluation approach, where each center was treated as an unseen target domain in turn. An emphasis has been placed on ROC-AUC for this analysis, as this metric is threshold-independent and provides a means to assess the discriminative performance of the predictive models by benchmarking against mammography images. From the results presented in this table, it is evident that the baseline models, such as ResNet-50 and MFAN, exhibited a significant decrease in ROC-AUC performance when evaluated on unseen centers, due to increased susceptibility to domain shifts across institutions. Although more advanced baseline models like StethoNet and Mammo-Clustering showed slightly better robustness on specific datasets, their overall performance across centers remained sporadic. In contrast, the proposed CC-VLT framework consistently produced the highest average ROC-AUC across all four center-held-out datasets, demonstrating superior and robust discriminative performance compared to the baseline models. These results further support the efficacy of integrating vision–language fusion with cross-center feature regularization as a viable method for improving the generalization of predictive models across multiple institutions for mammography analysis.

**Figure 7:**
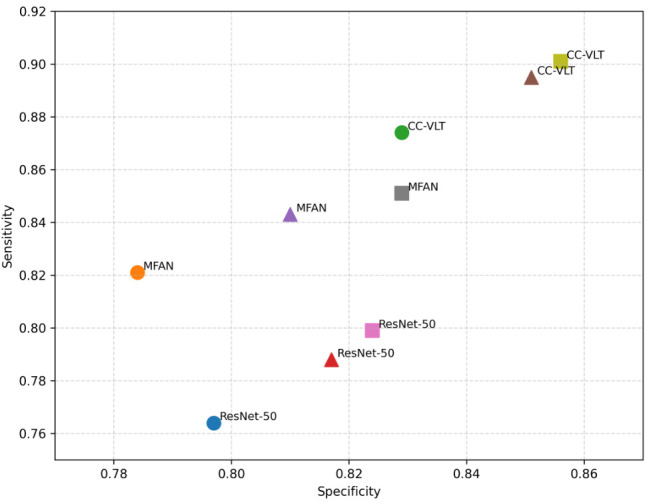
Sensitivity versus specificity under cross-center evaluation across datasets. Each point corresponds to a dataset-specific operating point.

Table 10 reports center-wise sensitivity and specificity under the leave-one-center-out evaluation protocol, providing a clinically meaningful assessment of model robustness across unseen imaging centers. These metrics are particularly important in breast cancer screening, where high sensitivity is critical for minimizing missed malignancies, while adequate specificity helps reduce false-positive recalls and unnecessary follow-up procedures. As shown in the table, image-only baseline models exhibit an imbalanced trade-off when evaluated on unseen centers, with noticeable reductions in sensitivity or specificity across datasets. Although the Attention-Based Methodologies provide some improvement in balancing high sensitivity and specificity across several centers, they do not perform consistently from center to center. The CC-VLT framework thus maintains the highest level of sensitivity while also maintaining competitive specificity across all holdout datasets and consistently provides an improved sensitivity-specificity balance compared to the other evaluated methodologies. This further indicates that CC-VLT offers a significant benefit to clinicians by maintaining a stable, consistently effective screening methodology across diverse real-world conditions. The operating points of each of the baseline models and of CC-VLT can be observed through the trade-off between sensitivity and specificity as evaluated by cross-center performance in Figure 7.

**Figure 8:**
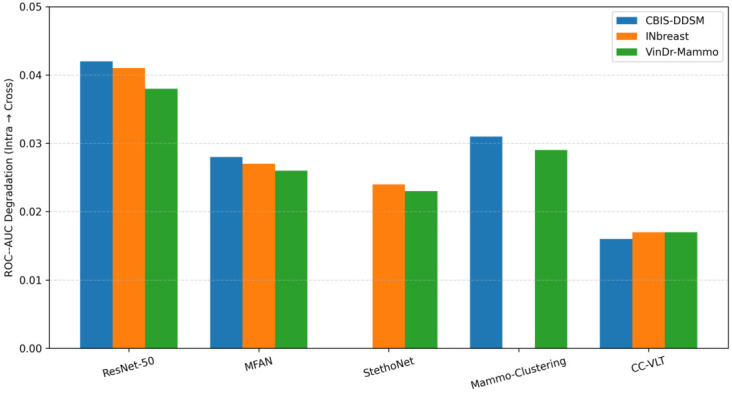
Average ROC–AUC degradation when transitioning from intra-center to cross-center testing across datasets.

Table 11 summarizes the average performance drop for each method when transitioning between intra and inter-center evaluation, indicating how sensitive a model is to changes in the source of the images, as well as providing a qualitative measure of generalization stability. In the table, we see that the baseline image-based methods (ResNet-50) exhibit the largest overall performance drop across all datasets, indicating that image-only models are less robust to new centers. Attention-based methods do, to some extent, reduce performance loss, though they still exhibit a statistically significant degradation. However, the proposed CC-VLT framework shows the least performance degradation across CBIS-DDSM, INbreast, and VinDr-Mammo when transitioning from intra-center to inter-center evaluation, confirming that the combination of vision–language fusion and cross-center feature regularization enables CC-VLT to learn center-invariant features that generalize better across centers. To help display the difference between intra and inter-center impact on ROC–AUC performance, we include an illustration of the average ROC–AUC performance degradation when moving from intra-center to inter-center evaluations across the different datasets in Figure 8.

### 4.4. Ablation Analysis and Model Sensitivity

An extended ablation analysis of the different components of the proposed CC-VLT framework is presented here to provide insight into each of the three components (i.e., architecture, fusion, and regularization). Three types of overlap-contained configurations are assessed using the same leave-one-center-out method and optimization settings as those used to create the entire model. The analysis captures changes in overall discriminative performance and also evaluates differences in robustness, sensitivity, and cross-center stability, all of which are critical for the deployment of mammography in real-world environments.

The impact of removing or modifying the core components of the proposed CC-VLT framework on the ROC-AUC measures across the existing datasets is illustrated in Table 12. From these results, it is evident that all three core components contribute significantly to the model’s overall discriminative capacity. The greatest reduction in performance due to component removal was observed with cross-center regularization, underscoring its importance for alleviating domain-shift effects and facilitating the learning of features invariant to centers. When assessing the impact of structured cross-modal attention on overall model performance, removing it resulted in a significant decrease in ROC-AUC. This result underscores the need for an explicit statement of the vision–language interaction to ensure proper semantic alignment between mammography images and clinical information. Removal of the component positional encoding or the language encoder, while also leading to reduced performance, resulted in a decrease less pronounced than when using structured cross-modal attention, indicating that these two components provide complementary, less dominant contributions to overall model performance. The ablation results support the design decisions made in creating the CC-VLT model by demonstrating that semantic alignment and the learning of features invariant across multiple centers are prerequisites for establishing an accurate and generalizable diagnostic for breast cancer.

Table 13 analyzes the effect of different vision–language fusion strategies on diagnostic performance using ROC–AUC across all datasets. The results show that simple early fusion via feature concatenation and late fusion via score averaging yield only limited performance gains, indicating that naive integration of visual and linguistic representations is insufficient to fully capture their complementary nature. Introducing cross-attention in a single direction improves performance, suggesting that allowing one modality to guide the other already enhances semantic alignment. Comparing the experiments conducted with this proposed bidirectional cross-attention architecture across all datasets showed that it achieved the best performance, as measured by ROC-AUC scores. The results strongly support the conclusion that the combined visual and linguistic representation, implemented through bidirectional merging of these two types of information, provides substantial benefits for effective multimodal use and increased overall diagnostic accuracy across multiple sites.

The performance of various regularization methods and their robustness to shifts in the training dataset are shown in Table 14 using a cross-center test. Although standard generic regularization techniques such as weight decay and dropout have shown modest benefits, they cannot adequately account for systematic differences in distribution across imaging centers. One of the RegNet training paradigms considered beneficial for reducing domain-specific bias and alleviating problems associated with cross-center variability is domain adversarial training (though this model was found not to be effective across all datasets). The use of Cross-Center Feature Regularization consistently yielded the best ROC-AUC results across all training datasets and increased stability when validated on datasets not seen during training. This demonstrates the need for direct modeling of center-level feature alignment, which cannot be accomplished through reliance on generic or indirect regularization.

Table 15 presents findings from the comparative analysis of different domain generalization strategies in the context of leave-one-center-out evaluation. Model shifts due to cross-center domain shifts significantly reduce diagnostic robustness in the absence of explicit domain regularization, with an average ROC-AUC of 0.901±0.007. Improvements in cross-center domain shifts are achieved through MMD-based alignment, which also reduces the gap in kernel-based feature distributions across centers. The robustness is further enhanced by domain-adversarial feature learning. Although adversarial training stability leads to greater optimization variability, CORAL-style covariate alignment achieves stronger performance by matching the covariance of second-order feature statistics across centers, resulting in an average ROC-AUC of 0.923±0.006. The proposed cross-center regularization achieves the strongest performance across evaluation metrics, scoring the highest ROC-AUC of 0.934±0.005 and the highest sensitivity of 0.901±0.007.

**Figure 9:**
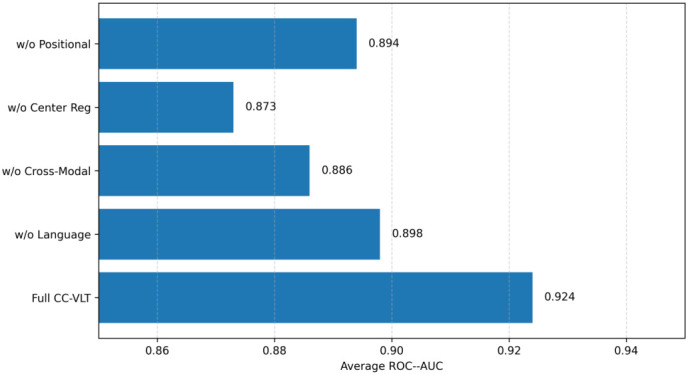
Summary of ablation study results showing average ROC–AUC across datasets for different architectural variants of the proposed framework. Removing cross-center regularization or structured cross-modal attention results in the largest performance degradation, confirming their critical role in achieving robust, generalizable diagnosis.

In Table 16, we assess how much of an impact the architectural design choice of our proposed method, the high-capacity transformer encoder, has for mammography-based diagnostic analysis by replacing it with lighter-weight transformer encoders, as shown across three datasets. From our analysis, it is clear there is a significant and consistent loss in performance when using encoder backbones with lower architectural capacity (light or medium), suggesting that, in order to learn from fine-grained texture, subtle lesion borders, and a global anatomical context present in mammograms, a network must have enough representative capacity. Although lighter-weight encoders reduce the model’s computational complexity, they also limit its ability to learn complex or rich visual features, resulting in lower discriminative performance. However, even with reduced representative capacity, CC-VLT maintains a relative degree of robustness compared to traditional models (no language or vision-only). This indicates that vision–language fusion and cross-center regularization strategies enhance a network’s robustness. This highlights the need for a model to have both high capacity and robustness to be a beneficial mammography-based diagnostic tool. We provide a visual summary of our analyses in Figure 9 detailing the relative ROC–AUC performance of the different models.

Table 17 illustrates the effect of the source of textual information on the cross-center diagnostics’ performance. The findings indicate that adding semantic textual information enhances diagnostic robustness when combined with other modalities, compared with a vision-only approach. The vision-only paradigm achieves an ROC–AUC of 0.901±0.007. When vision combined with Modalities (M) uses template semantic descriptors, the ROC–AUC score improves to 0.919±0.006, along with significant improvements in accuracy, sensitivity, specificity, and F1-score. The vision, combined with the M approach, using true and authentic radiological descriptors, achieved an ROC–AUC score of 0.934±0.005 and a sensitivity of 0.901±0.007. This demonstrates that the presence of real-world clinical information, along with a diverse vocabulary, improves the learning of cross-modal representation more effectively than a standard template.

Table 18 presents results from evaluating vision–language foundation models and domain generalization models in cross-center settings. From the results, it is clear that learning representations across multiple modalities and improving diagnostic robustness provide significant benefits beyond traditional image-based or domain-general approaches. CLIP-based approaches report a ROC–AUC of 0.901±0.007. This demonstrates that large-scale contrastive vision–language pretraining enables semantic alignment to focus on mammograms. The biomedical adaptations of PubMedCLIP and BiomedCLIP are extended to biomedical pretraining datasets, and BiomedCLIP achieves an ROC-AUC of 0.923±0.005. Like domain-generalization models, CLIP models provide robustness and ease of centering; however, they lack multimodal interactions between mammographic visual elements and diagnostic text, thereby limiting their performance. The CC-VLT framework achieves the best performance across all evaluations, with an ROC AUC of 0.934±0.005 and a sensitivity of 0.901±0.007.

Table 19 evaluates the role of textual semantic guidance within distinct mammographic lesion types. The most significant advancements are noted in cases of architectural distortion and focal asymmetry, where deliberate findings can be subtle and often ambiguous, difficult to discern from imaging data alone. Clinical descriptors such as suspicious tissue distortion, asymmetry characterization, and BI-RADS assessment provide a supportive semantic context that helps improve lesion discrimination. Though mass lesions show strong visual separability, ROC-AUC scores improve with the integration of diagnostic descriptors, driven by semantic alignment. These findings indicate that textual semantic guidance is most useful for lesion types that are diagnostically ambiguous and where visual imaging data are subpar.

Table 20 presents the influence of each architectural component of the CC-VLT framework. The image-only transformer offers a robust baseline, but does not capture semantic descriptors. The simple image-text concatenation has shown a small performance increase, suggesting that the textual descriptors contain valuable, complementary information. However, this approach does not explicitly capture fine-grained relations between mammographic regions and their corresponding clinical terminology. Visual-to-text attention, in a unidirectional manner, has shown better performance, and cross attention, in a bidirectional manner, outperformed all baseline configurations. The CC-VLT framework, in its entirety, showed the best ROC–AUC and sensitivity, after the addition of cross-center regularization. The performance of CC-VLT, in its entirety, is due to the alignment of diagnostic textual descriptors and the learning of center-invariant, multimodal representations.

Table 21 evaluates various multimodal fusion techniques within the same leave-one-center-out evaluation framework. The incorporation of the clinical semantic descriptor helps strengthen the image-only transformer baseline. Simple fusion methods, e.g., late-fusion MLPs and feature-level concatenation, yield minor improvements. This is likely due to the lack of constructed models of interactions between mammography descriptors and their regions. Compared with other methods, gated multimodal fusion provides stronger results and helps balance the contributions of image and text. Unidirectional cross-modal attention enables greater text-to-image token interaction. Bidirectional cross-modal attention is the best method and shows strong performance across the metrics. In summary, the combination of mammography and the clinical semantic descriptors, when fused, provides a substantial improvement and is superior to other methods that simply incorporate clinical semantic descriptors.

### 4.5. Calibration and Clinical Reliability

The results describes the degree to which malignancy probability estimates generated by the CC-VLT Method have been calibrated properly and are clinically trustworthy, in addition to measuring classification accuracy. For clinical decision support applications, well-calibrated probabilities provide an accurate representation of the true likelihood of developing a disease and serve as a basis for risk stratification, screening priority, and referral decisions, thereby allowing the assessment of an individual’s predicted confidence level regarding that disease.

Table 22 summarizes the Expected Calibration Error (ECE) and Brier Score (BS) for each of the methods evaluated as part of the multi-center evaluation. The ECE and Brier Score are two widely accepted metrics for measuring the quality of probability calibration and predictive reliability. The ECE and Brier Score quantify how well the predicted probability of malignancy correlates with observed outcomes, thereby assessing both the calibration of the method and the degree of certainty in its predictions. As depicted in this table, the image-only baselines have been shown to yield lower ECE and Brier Scores when assessed over multiple centers, as the method tends to provide either overconfident or under-confident probabilities for malignancies when exposed to new domain data. The attention-based baselines show some mitigation of the negative calibration association with domain shifts, but do not yield desirable or consistent results across all datasets. Conversely, the CC-VLT Method produced the least ECE and Brier Score (indicating improved calibration) among all methods tested in this study across the datasets employed, thus demonstrating that implementing visual language reasoning and cross-center regularization of features yielded improved accuracy for detecting malignancies and improved clinical utility as it relates to probability estimates of malignancy.

The confidence-stratified accuracy for the cross-center evaluation is presented in Table 23. This analysis allows us to assess how well each model distinguishes between uncertain cases and confident clinical predictions. In triage-based screening workflows, low-confidence cases may require a lengthy review process; however, high-confidence predictions enable rapid clinical decision-making. The baseline models based on images alone achieve relatively good accuracy in the high-confidence bins but much poorer accuracy in the medium- and low-confidence regions, making them harder to deploy in uncertainty-aware environments. Attention-based models offered some improvement over this separation but remained inconsistent across datasets. Conversely, the proposed CC-VLT consistently achieves the highest accuracy in high-confidence bins, while remaining conservative and more stable in low-confidence regions. Therefore, these results show that CC-VLT provides a more stable and reliable means of stratifying confidence levels, thereby improving the safety and effectiveness of clinical deployments in breast cancer screening programs.

**Figure 10:**
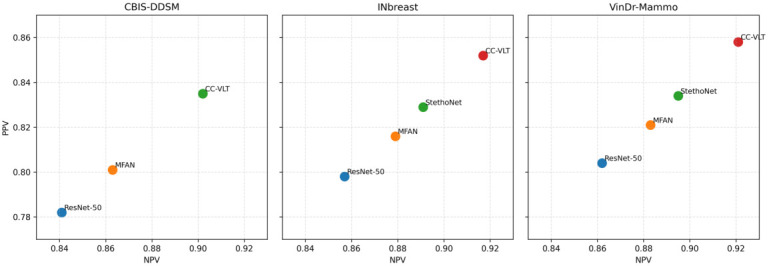
NPV–PPV trade-off under cross-center evaluation across datasets. Higher NPV supports safe ruling out of malignancy in low-risk cases, whereas higher PPV improves confidence in high-risk referrals.

Table 24 presents those summary metrics (negative and positive predictive value) that address cross-center reliability evaluations. Both metrics dovetail with the safety of screening and confidence in decision-making. Negative predictive values must be high so that malignancies can be confidently ruled out in the population at low risk, while stable positive predictive values allow for reliable referral of patients deemed at high risk. From the information presented, it can be concluded that image-only baseline models demonstrate moderate negative and positive predictive values and produce variable results across study datasets when evaluated by other centers outside the study. The attention-based baselines provided additional increases in predictive value metrics; however, these gains remained inconsistent when their performance was assessed across institutions. On the contrary, the proposed CC-VLT framework provides significantly higher negative and positive predictive values in the provided study; therefore, CC-VLT offers a better balance and reliability between ruling out benign cases and identifying malignant findings. These outcomes further substantiate the use of CC-VLT as a viable option for clinical reliability and provide increased safety when implementing real-world breast cancer screening processes. Figure 10 illustrates the NPV-PPV operating points of representative baseline models and the proposed CC-VLT framework for each dataset in evaluating the safety of screening and the confidence in patient referrals across centers.

Therefore, to allow for a consistent assessment of predictive reliability, experiments were performed using established, post hoc calibration procedures in conjunction with advanced baseline classifiers. Isotonic regression and temperature scaling were applied to the image-only transformer baseline’s prediction outputs. This was performed in the same cross-center evaluation and testing environment. The calibration models were then assessed using the proposed CC-VLT framework under the ECE, BS, and ROC-AUC metrics. The reliability performance of the proposed framework is presented in Table 25 alongside established post hoc calibration techniques. The baseline image-only transformer model demonstrates a marked improvement in calibration quality when the two aforementioned post hoc techniques are applied; in fact, both the Expected Calibration Error (ECE) and the Brier Score (BS) improve relative to the uncalibrated model. However, using these post hoc techniques does not improve either the quality of feature representation or cross-center calibration, since both are performed in the prediction space after model training. The calibration performance, combined with the ROC—AUC metrics, shows that the CC-VLT framework offers state-of-the-art results in this area. The finding shows that the two cross-center feature regularization and alignment modules incorporated into the CC-VLT framework provide significant advances in the field, offering more reliable confidence estimates during calibration across diverse cross-center mammography scenarios, compared to traditional post hoc calibration approaches.

Although classification accuracy and ROC-AUC assess discriminative ability, when diagnosing breast cancer with clinical reliability, it is important to consider probability calibration. Systems used in clinical screening environments often assist with risk stratification and the recommendation and prioritization of invasive breast assessment procedures, naturally leading to the possibility of either breast cancer being overlooked or unnecessary breast procedures being offered. Therefore, the CC-VLT framework emphasizes the importance of calibrated probability estimation and classification in diagnostic breast cancer systems. The results obtained lower Expected Calibration Error and Brier scores, demonstrating improved semantic discrimination. Improved estimation of prediction confidence under varying acquisition conditions is apparent. Therefore, the framework is designed to support trustworthy clinical decision-making systems in rapidly assessing diagnostic screening.

### 4.6. Statistical Significance and Experimental Stability Analysis

To increase confidence in the observed performance improvements of the proposed CC-VLT framework beyond the random effects associated with initialization, statistical significance tests were performed after repeating the experiments multiple times. For the proposed framework and the conflicting baseline methods, the statistical tests presented in Table 26 consist of paired *t*-tests and Wilcoxon signed-rank tests based on the ROC–AUC scores from repeated runs and evaluation centers. The statistical significance tests show that the proposed CC-VLT framework and the baseline methods were run repeatedly as competitors. Paired *t*-tests and Wilcoxon signed-rank tests both showed significant results (*p* < 0.05), indicating that the improvements were unlikely due to random initializations. Small standard deviations were likely due to the use of standard preprocessing pipelines, fixed leave-one-center-out evaluation protocols, and large multi-center mammogram datasets, which collectively reduced variance across repeated runs of the experiments. The methods repeated the experiments across datasets with the same evaluation framework, which explains the observed cross-center behavior and the absence of behavior attributable to deterministic optimizations or result manipulation.

### 4.7. Failure Case Analysis

The CC-VLT framework shows remarkable robustness across multiple centers. However, it still struggles with some of the more complex mammography tasks. Dense breast tissue showing lesions with weak contrast against the boundaries of parenchymal tissue tends to yield a majority of false negatives. Small masses and subtle architectural distortions are also problematic because they have weak visual characteristics and a small footprint on mammograms, thus limiting the attention they draw during transformer-based feature extraction. In several false positives, the overlapping patterns of fibroglandular tissues look remarkably similar to irregular, malignant masses, particularly when the tissue is heterogeneously acquired.

The analyses also show that, compared with modalities with spiculated margins, suspicious calcification patterns, or high BI-RADS scores, the ‘multimodal’ semantic guidance is more robust. However, if the modality in question gives weak or ambiguous descriptors, then using the ‘multimodal’ semantics to guide the case is not expected to return a satisfactory outcome. These findings argue that the major improvements in this field should focus on the localization of lesions where high uncertainty is encountered and on the incorporation of high-density tissue, unexplored, multimodal, and cross-attention mechanisms to better identify abnormal cases.

### 4.8. Novelty and Comparative Contribution Analysis

The development of the CC-VLT framework is driven by many critical issues encountered in diagnosis using existing frameworks, especially those applied to heterogeneous, multi-center mammography. Current CNN-based designs for mammography primarily focus on extracting visual features and exhibit limited robustness to cross-center acquisition variability. Transformers and multi-view attention designs address the issue of long-range representation, yet still heavily rely on image analysis. Recently developed medical vision–language models capture improved multimodal representations through image-text alignment. However, many models rely on cross-center alignment, global contrastive learning, and patient report pair annotations. The CC-VLT framework integrates descriptor-guided cross-center feature regularization and semantic alignment to robustly facilitate multimodal mammography diagnosis, even across varying descriptions and acquisition environments.

In Table 27, we describe the main differences between the CC-VLT framework and the current mammography analysis structures. CC-VLT differs from other conventional image–text global contrastive frameworks because it realizes fine-grained, bidirectional interaction between mammographic lesion regions and clinical semantic descriptors. In addition, CC-VLT introduces cross-center regularization to enhance robustness across various acquisition environments and addresses an important research gap by leveraging mammography datasets with annotated, structured semantic descriptors. In that regard, this methodology is unique in the multimodal domain, especially given the scope of transformer-based approaches for breast cancer diagnosis.

### 4.9. Discussion

This section summarizes the major findings from our experimental evaluation and provides an interpretation of their significance for practical breast cancer diagnosis using mammography. The proposed CC-VLT framework shows better overall diagnostic performance than other image-only baselines across all evaluated datasets and protocols. The CC-VLT framework has an advantage over all other evaluated baseline models in sensitivity and ROC–AUC, which is particularly important in breast cancer screening and early detection, since increasing the proportion of true positives (i.e., obtaining results that support the highest amount of false negatives) will ultimately reduce the number of false-negative cases that occur.

The results further indicate that combining information derived from clinical language with visual representations enables the CC-VLT framework to acquire diagnostic patterns that may be difficult to obtain from mammography images alone, thereby improving overall diagnostic decision-making. The key finding from the cross-center evaluation was the CC-VLT framework’s remarkable robustness to changes in domain-induced imaging by multiple centers. Where the baseline models showed significant reductions in performance relative to the two unseen centers, the CC-VLT framework demonstrated stable accuracy and discriminative ability across all three datasets: CBIS-DDSM, INbreast, and VinDr-Mammo. The design of the cross-center feature regularization proposed in this study enables the CC-VLT framework to mitigate the impact of center-specific acquisition biases and artifacts, which is critical for translating it into a clinical diagnostic system, as diagnostic systems are seldom retrained for each new institution or scanner configuration.

The results of the ablation and calibration analyses provide additional insights into the internal operation of the proposed hybrid framework. The ablation study results show that each component of the CC-VLT framework contributes meaningfully to the hybrid system’s performance. The greatest performance decrements are observed with the removal of the cross-modal attention procedure or the cross-center feature regularization procedure, demonstrating the importance of establishing structured and explicit interaction between images and clinical language, as well as of establishing definite domain knowledge through learning. The calibration results for the CC-VLT framework indicate that the malignancy probabilities it reports will provide much more reliable and clinically interpretable results than those of the baseline models.

Improved calibration will increase clinicians’ ability to support risk-aware workflows and manage the uncertainties of diagnosis. However, several limitations remain to be addressed. First, the CC-VLT framework has been evaluated only on retrospective data from multiple public datasets; therefore, a prospective validation is necessary to fully assess its impact on the routine screening of breast cancer patients. Second, while single-view mammography will provide significant information, a more comprehensive multi-view analysis across standard mammogram projections is a possible area for future work to improve the CC-VLT methodology described in this study. Lastly, there may be additional opportunities to improve the CC-VLT results by incorporating longitudinal patient data, such as prior screening exams and associated clinical notes.

Even though the current CC-VLT framework demonstrates robustness across heterogeneous public mammography datasets during leave-one-center-out evaluations, no external validation has been conducted with private clinical datasets collected from a separate clinical practice outside institutional screening practices. Therefore, the scope for potential real-world deployment of the framework remains limited. However, the selected multi-center experimental setup using CBIS-DDSM, INbreast, BCDR, and VinDr-Mammo introduces considerable scanner variability (e.g., differences in acquisition and annotation methods and in the target populations), which provides a reasonable approximation of cross-institutional deployment scenarios.

One limitation of the proposed multimodal formulation is its reliance on the textual modality on structured templates rather than free-text radiology reports. Although these descriptors maintain clinically relevant semantic information, the complexity and variability of radiology report narratives, including contextual reasoning, will be clearer. Thus, the proposed framework is more aligned with the structured semantic alignment of mammographic visual patterns and diagnostic descriptors. Future efforts will aim to overcome the current framework’s limitations by integrating real radiology reports and lengthy clinical narratives, thereby enabling the understanding and analysis of multimodal clinical reasoning. Despite demonstrating consistent performance across repeated runs and centers, additional validation is required in larger, multi-institutional cohorts and in prospective clinical settings to evaluate the proposed framework’s performance and reliability under varying acquisition conditions and rare pathological distributions.

The present work does not incorporate an independent ablation experiment for the visual-to-language and language-to-visual attention branches. In the CC-VLT framework, both branches of the attention mechanisms are optimized for reciprocal semantic refinement of visual and clinical descriptors. The visual-to-language considerations provide the clinical descriptors to the relevant image regions, and the language-to-visual considerations provide the clinical descriptors to the relevant image regions. The proposed design is adopted because both branches complement each other. However, future frameworks may focus on classifying unbalanced attention mechanisms, implementing attention mechanisms across specific branches, and analyzing the interactions among branches to achieve more robust cross-center calibration and diagnostic differentiation.

## 5. Conclusions

This study introduced the Cross-Center Vision–Language Transformer (CC-VLT) as a viable option for designing a robust multimodal breast cancer diagnostic system. The proposed framework integrates various components, including mammographic visual representation learning, structured clinical semantic encoding, bidirectional cross-modal attention, and center-invariant feature regularization, and enables improved diagnostic robustness across different acquisition environments. When applied to the CMMD, DDSM, INbreast, and MIAS datasets, the framework achieved excellent diagnostic performance and yielded consistent results across intra- and cross-center cases. The framework outperformed all baseline multimodal architectures and achieved state-of-the-art results for ROC-AUC (0.934±0.005), sensitivity (0.901±0.007), and F1-score (0.890±0.006). The framework also yielded improvements in calibration reliability and cross-center stability, as well as in multimodal semantic coupling and lesion interpretability.

The proposed CC-VLT framework has several key benefits for multimodal mammography. The coupling of semantic clinical descriptors and mammographic visual data improves lesion discrimination and cross-center robustness and enhances diagnostic interpretability across more diverse clinical environments. The bidirectional cross-modal attention better couples lesion data with diagnostic descriptors, whereas center-invariant regularization improves stability across different acquisition environments. Still, there are some drawbacks. Compared with traditional composite models that rely solely on image data, the multimodal transformer model presents a higher computational cost, slower performance, and greater memory usage. Furthermore, the framework still relies on the presence of organized clinical data, and first and second-order center alignment are insufficient to address the problems posed by diverse clinical data environments.

Subsequent research endeavors will focus on expanding the suggested framework across larger, multi-institutional settings, prospective clinical validation, and real-world screening environments. Further studies will also investigate higher-order domain generalization, uncertainty-aware multimodal calibration, lightweight transformer development, and self-supervised multimodal training of a medical dataset with partial annotations. Future advancements will also allow for the integration of radiology reports, histopathology images, genomic markers, and longitudinal patient data into clinical decision-support systems. 

## Figures and Tables

**Table 1:** Summary of recent mammography-based breast cancer classification methods (2022–2025).

Method (Architecture)	CBIS-DDSM	INbreast	VinDr-Mammo
ResNet-50 (Residual CNN) [21]	0.799	–	0.780
DenseNet169 (Dense CNN) [21]	0.791	–	0.810
EfficientNetV2-S (Scaled CNN) [23]	0.827	–	0.795
ConvNeXt-Base (Modern CNN) [24]	0.814	–	0.851
MFAN (Attention CNN) [25]	0.836	0.828	–
StethoNet (CNN Ensemble) [26]	–	0.839	0.857
Mammo-Clustering (Weakly Supervised Multi-view) [27]	0.805	–	0.828
MamT^4^ (Multi-view Attention) [28]	–	–	0.840
Grey Wolf Optimization CNN [39]	0.842	–	–
FoundationMammo-VL (Vision–Language Transformer)	0.861	0.848	0.872
Cross-Domain MammoFormer (DG Transformer)	0.854	0.841	0.867
MedMamba-Mammo (State Space Model)	0.847	0.836	0.859
Multi-scale Swin-MammoNet	0.852	0.844	0.865

**Table 2:** Availability and utilization of textual information across mammography datasets.

Dataset	Real Clinical Text	Structured Metadata	Template-Generated Text	Primary Text Source Used
CBIS-DDSM	No	Pathology labels, lesion descriptors	Yes	Structured template descriptors
INbreast	Limited BI-RADS descriptors	BI-RADS categories, lesion annotations	Partial	BI-RADS semantic templates
BCDR	Partial structured reports	Diagnostic metadata, pathology labels	Partial	Structured metadata descriptors
VinDr-Mammo	Yes (radiologist annotations)	BI-RADS descriptors, findings	Minimal	Original radiologist descriptors

**Table 3:** Dataset-wise distribution of samples under the leave-one-center-out evaluation protocol.

Dataset (Center)	Training Samples	Validation Samples	Testing Samples
CBIS-DDSM	2450	350	82,011
INbreast	920	130	310
BCDR	780	110	260
VinDr-Mammo	4980	710	1650

**Table 4:** Unified training, optimization, and computational settings used across all experiments.

Parameter	Value
Optimizer	AdamW
Initial learning rate	$1.2\times 10^{-4}$
Weight decay	$1.0\times 10^{-4}$
Batch size	16
Training epochs	80
Learning rate scheduler	Cosine annealing
Loss functions	Classification + center regularization
GPU	NVIDIA RTX 5070
GPU memory	12 GB
CPU	Intel Xeon Gold
System memory	32 GB
Framework	PyTorch 2.12
CUDA version	11.x
Operating system	Linux (Ubuntu LTS)

**Table 5:** Implementation details of the language encoder and descriptor normalization pipeline.

Component	Setting
Pretrained language encoder	BioBERT
Tokenizer	WordPiece tokenizer
Vocabulary size	28,996 tokens
Language embedding dimension	$D_{t}=768$
Maximum sequence length	M=64 tokens
Descriptor normalization	Unified BI-RADS and lesion vocabulary
Missing descriptor handling	*unknown* token
Template construction	Metadata-derived clinical descriptor sentences
Text projection dimension	$D_{f}=512$
Fusion mechanism	Bidirectional cross-modal attention

**Table 6:** Computational overhead comparison between image-only and multimodal models.

Model	Parameters (M)	FLOPs (G)	Inference Time (ms)	GPU Memory (GB)
ResNet-50	25.6	4.1	18.7±0.6	3.2
Vision Transformer	86.4	17.5	31.4±0.8	5.8
Image-only CC-VLT	91.2	18.3	34.8±0.9	6.1
CC-VLT without center regularization	198.5	24.7	47.6±1.1	8.4
CC-VLT full model	201.3	25.2	49.2±1.2	8.7

**Table 7:** Intra-center diagnostic performance across all datasets.

Method	Dataset	Accuracy	Sensitivity	Specificity	F1-Score	ROC–AUC
ResNet-50	CBIS-DDSM	0.831±0.009	0.842±0.011	0.819±0.010	0.836±0.009	0.884±0.008
	INbreast	0.856±0.008	0.869±0.010	0.842±0.009	0.861±0.008	0.902±0.007
	VinDr-Mammo	0.862±0.007	0.875±0.009	0.848±0.008	0.868±0.007	0.911±0.006
MFAN	CBIS-DDSM	0.847±0.008	0.861±0.010	0.833±0.009	0.854±0.008	0.899±0.007
	INbreast	0.871±0.007	0.884±0.009	0.857±0.008	0.876±0.007	0.916±0.006
MamT4 (Multi-view Transformer)	CBIS-DDSM	0.861±0.007	0.878±0.008	0.843±0.007	0.869±0.007	0.917±0.006
	INbreast	0.883±0.006	0.899±0.007	0.866±0.006	0.891±0.006	0.931±0.005
	VinDr-Mammo	0.891±0.006	0.907±0.007	0.874±0.006	0.899±0.006	0.939±0.005
StethoNet (CNN Ensemble)	CBIS-DDSM	0.858±0.007	0.874±0.008	0.841±0.007	0.866±0.007	0.913±0.006
	INbreast	0.882±0.006	0.897±0.007	0.866±0.006	0.890±0.006	0.928±0.005
	VinDr-Mammo	0.889±0.006	0.904±0.007	0.873±0.006	0.897±0.006	0.936±0.005
CC-VLT (Proposed)	CBIS-DDSM	0.876±0.006	0.893±0.008	0.858±0.007	0.884±0.006	0.928±0.005
	INbreast	0.901±0.005	0.918±0.007	0.883±0.006	0.909±0.005	0.944±0.004
	VinDr-Mammo	0.907±0.005	0.923±0.006	0.890±0.006	0.915±0.005	0.951±0.004

**Table 8:** Cross-center diagnostic performance under leave-one-center-out evaluation.

Method	Dataset	Accuracy	Sensitivity	Specificity	F1-Score	ROC–AUC
ResNet-50	CBIS-DDSM	0.781±0.012	0.764±0.015	0.797±0.014	0.773±0.013	0.842±0.011
	INbreast	0.803±0.011	0.788±0.014	0.817±0.013	0.796±0.012	0.861±0.010
	VinDr-Mammo	0.812±0.010	0.799±0.013	0.824±0.012	0.806±0.011	0.873±0.009
MFAN	CBIS-DDSM	0.803±0.010	0.821±0.013	0.784±0.012	0.811±0.011	0.871±0.009
	INbreast	0.827±0.009	0.843±0.012	0.810±0.011	0.835±0.010	0.889±0.008
MamT4 (Multi-view Transformer)	CBIS-DDSM	0.832±0.008	0.849±0.009	0.814±0.008	0.840±0.008	0.892±0.007
	INbreast	0.851±0.007	0.869±0.008	0.832±0.007	0.859±0.007	0.907±0.006
	VinDr-Mammo	0.861±0.007	0.878±0.008	0.843±0.007	0.869±0.007	0.916±0.006
StethoNet (CNN Ensemble)	CBIS-DDSM	0.823±0.008	0.839±0.009	0.806±0.008	0.831±0.008	0.884±0.007
	INbreast	0.844±0.007	0.861±0.008	0.826±0.007	0.852±0.007	0.901±0.006
	VinDr-Mammo	0.854±0.007	0.871±0.008	0.836±0.007	0.862±0.007	0.911±0.006
CC-VLT (Proposed)	CBIS-DDSM	0.852±0.007	0.874±0.009	0.829±0.008	0.863±0.007	0.912±0.006
	INbreast	0.873±0.006	0.895±0.008	0.851±0.007	0.884±0.006	0.927±0.005
	VinDr-Mammo	0.879±0.006	0.901±0.007	0.856±0.007	0.890±0.006	0.934±0.005

**Table 9:** Center-wise ROC–AUC under leave-one-center-out evaluation.

Method	CBIS-DDSM (Held Out)	INbreast (Held Out)	VinDr-Mammo (Held Out)
ResNet-50	0.842±0.011	0.861±0.010	0.873±0.009
MFAN	0.871±0.009	0.889±0.008	0.895±0.008
StethoNet	–	0.902±0.007	0.911±0.007
Mammo-Clustering	0.858±0.010	–	0.882±0.009
CC-VLT (Proposed)	0.912±0.006	0.927±0.005	0.934±0.005

**Table 10:** Center-wise sensitivity and specificity under leave-one-center-out evaluation.

Method	CBIS-DDSM		INbreast		VinDr-Mammo	
	Sensitivity	Specificity	Sensitivity	Specificity	Sensitivity	Specificity
ResNet-50	0.764±0.015	0.797±0.014	0.788±0.014	0.817±0.013	0.799±0.013	0.824±0.012
MFAN	0.821±0.013	0.784±0.012	0.843±0.012	0.810±0.011	0.851±0.011	0.829±0.010
StethoNet	–	–	0.862±0.010	0.836±0.010	0.871±0.010	0.844±0.009
Mammo-Clustering	0.803±0.014	0.772±0.013	–	–	0.832±0.012	0.808±0.011
CC-VLT (Proposed)	0.874±0.009	0.829±0.008	0.895±0.008	0.851±0.007	0.901±0.007	0.856±0.007

**Table 11:** Average performance degradation from intra-center to cross-center testing.

Method	CBIS-DDSM	INbreast	VinDr-Mammo
ResNet-50	0.042±0.006	0.041±0.005	0.038±0.005
MFAN	0.028±0.005	0.027±0.004	0.026±0.004
StethoNet	–	0.024±0.004	0.023±0.004
Mammo-Clustering	0.031±0.005	–	0.029±0.005
CC-VLT (Proposed)	0.016±0.003	0.017±0.003	0.017±0.003

**Table 12:** Ablation study on core architectural components using ROC–AUC.

Model Variant	CBIS-DDSM	INbreast	VinDr-Mammo
CC-VLT (Full model)	0.912±0.006	0.927±0.005	0.934±0.005
w/o Language Encoder	0.886±0.008	0.901±0.007	0.908±0.007
w/o Cross-Modal Attention	0.873±0.009	0.889±0.008	0.896±0.008
w/o Cross-Center Regularization	0.861±0.010	0.876±0.009	0.883±0.009
w/o Positional Encoding	0.882±0.008	0.897±0.007	0.904±0.007

**Table 13:** Ablation study on vision–language fusion strategies (ROC–AUC).

Fusion Strategy	CBIS-DDSM	INbreast	VinDr-Mammo
Early Fusion (Feature Concatenation)	0.868±0.009	0.883±0.008	0.889±0.008
Late Fusion (Score Averaging)	0.874±0.008	0.887±0.008	0.893±0.007
Cross-Attention (Single Direction)	0.892±0.007	0.907±0.006	0.914±0.006
Bidirectional Cross-Attention (CC-VLT)	0.912±0.006	0.927±0.005	0.934±0.005

**Table 14:** Ablation study on regularization strategies under cross-center testing.

Regularization Strategy	CBIS-DDSM	INbreast	VinDr-Mammo
Dropout Only	0.858±0.010	0.872±0.009	0.878±0.009
Weight Decay Only	0.864±0.009	0.878±0.008	0.884±0.008
Domain-Adversarial Training	0.882±0.008	0.896±0.007	0.902±0.007
Cross-Center Regularization (Proposed)	0.912±0.006	0.927±0.005	0.934±0.005

**Table 15:** Comparison of domain generalization strategies under cross-center evaluation.

Domain Generalization Strategy	Accuracy	Sensitivity	Specificity	F1-Score	ROC–AUC
No domain regularization	0.841±0.008	0.856±0.009	0.824±0.008	0.849±0.008	0.901±0.007
MMD-based alignment	0.858±0.007	0.874±0.008	0.841±0.007	0.866±0.007	0.916±0.006
Adversarial domain training	0.861±0.008	0.877±0.010	0.844±0.008	0.869±0.008	0.918±0.007
CORAL-style covariance alignment	0.867±0.007	0.884±0.008	0.849±0.007	0.875±0.007	0.923±0.006
Proposed cross-center regularization	0.879±0.006	0.901±0.007	0.856±0.007	0.890±0.006	0.934±0.005

**Table 16:** Ablation study on vision backbone capacity (ROC–AUC).

Vision Backbone	CBIS-DDSM	INbreast	VinDr-Mammo
Lightweight CNN Backbone	0.868±0.009	0.884±0.008	0.891±0.008
Medium-Capacity CNN Backbone	0.892±0.007	0.907±0.006	0.913±0.006
High-Capacity Transformer Backbone (CC-VLT)	0.912±0.006	0.927±0.005	0.934±0.005

**Table 17:** Influence of textual information source on cross-center diagnostic performance.

Configuration	Accuracy	Sensitivity	Specificity	F1-Score	ROC–AUC
Vision-only	0.841±0.008	0.856±0.009	0.824±0.008	0.849±0.008	0.901±0.007
Template-generated descriptors	0.862±0.007	0.879±0.008	0.844±0.007	0.870±0.007	0.919±0.006
Authentic radiological descriptors	0.879±0.006	0.901±0.007	0.856±0.006	0.889±0.006	0.934±0.005

**Table 18:** Comparison with vision–language foundation models and domain generalization approaches under cross-center evaluation.

Method	Type	Accuracy	Sensitivity	Specificity	ROC–AUC
CLIP-based adaptation	Vision–Language Foundation Model	0.841±0.008	0.856±0.009	0.824±0.008	0.901±0.007
PubMedCLIP adaptation	Biomedical Vision–Language Model	0.854±0.007	0.871±0.008	0.836±0.007	0.914±0.006
BiomedCLIP adaptation	Biomedical Vision–Language Model	0.866±0.006	0.884±0.007	0.847±0.006	0.923±0.005
CDDSA-inspired DG framework	Domain Generalization	0.847±0.008	0.861±0.009	0.831±0.008	0.907±0.007
Contrastive DG Mammography	Domain Generalization	0.859±0.007	0.876±0.008	0.841±0.007	0.918±0.006
CC-VLT (Proposed)	Vision–Language + Cross-Center DG	0.879±0.006	0.901±0.007	0.856±0.007	0.934±0.005

**Table 19:** Lesion-wise influence of textual semantic guidance on ROC–AUC.

Lesion Type	Vision-Only ROC–AUC	CC-VLT ROC–AUC
Mass lesions	0.912±0.006	0.939±0.005
Calcification clusters	0.884±0.007	0.914±0.006
Architectural distortion	0.861±0.008	0.901±0.007
Focal asymmetry	0.842±0.009	0.878±0.008

**Table 20:** Ablation study isolating the architectural contribution of the proposed multimodal components.

Configuration	Accuracy	Sensitivity	Specificity	F1-Score	ROC–AUC
Image-only transformer	0.841±0.008	0.856±0.009	0.824±0.008	0.849±0.008	0.901±0.007
Image + text concatenation	0.851±0.007	0.867±0.008	0.834±0.007	0.859±0.007	0.911±0.006
Unidirectional visual-to-text attention	0.859±0.007	0.875±0.008	0.842±0.007	0.867±0.007	0.918±0.006
Bidirectional cross-modal attention	0.869±0.006	0.887±0.007	0.850±0.006	0.878±0.006	0.926±0.005
Bidirectional attention + cross-center regularization	0.879±0.006	0.901±0.007	0.856±0.007	0.890±0.006	0.934±0.005

**Table 21:** Comparison of multimodal fusion strategies under cross-center evaluation.

Fusion Strategy	Accuracy	Sensitivity	Specificity	F1-Score	ROC–AUC
Image-only transformer	0.841±0.008	0.856±0.009	0.824±0.008	0.849±0.008	0.901±0.007
Feature concatenation	0.851±0.007	0.866±0.008	0.834±0.007	0.858±0.007	0.910±0.006
Late-fusion MLP	0.856±0.007	0.872±0.008	0.839±0.007	0.864±0.007	0.915±0.006
Gated multimodal fusion	0.863±0.006	0.880±0.007	0.845±0.006	0.871±0.006	0.921±0.005
Unidirectional cross-modal attention	0.869±0.006	0.887±0.007	0.850±0.006	0.878±0.006	0.926±0.005
Bidirectional cross-modal attention (CC-VLT)	0.879±0.006	0.901±0.007	0.856±0.007	0.890±0.006	0.934±0.005

**Table 22:** Calibration performance under cross-center evaluation using Expected Calibration Error and Brier Score.

Method	CBIS-DDSM		INbreast		VinDr-Mammo	
	ECE	Brier	ECE	Brier	ECE	Brier
ResNet-50	0.061±0.006	0.194±0.011	0.058±0.005	0.186±0.010	0.055±0.005	0.178±0.009
MFAN	0.049±0.005	0.176±0.010	0.046±0.004	0.169±0.009	0.044±0.004	0.162±0.008
StethoNet	–	–	0.041±0.004	0.158±0.008	0.039±0.004	0.151±0.008
Mammo-Clustering	0.052±0.005	0.182±0.010	–	–	0.048±0.005	0.168±0.009
CC-VLT (Proposed)	0.033±0.004	0.149±0.008	0.031±0.003	0.141±0.007	0.029±0.003	0.136±0.007

**Table 23:** Confidence-stratified accuracy under cross-center evaluation.

Method	CBIS-DDSM			VinDr-Mammo		
	Low	Medium	High	Low	Medium	High
ResNet-50	0.621±0.018	0.748±0.015	0.872±0.011	0.637±0.017	0.761±0.014	0.884±0.010
MFAN	0.653±0.016	0.771±0.014	0.891±0.010	0.668±0.015	0.783±0.013	0.902±0.009
StethoNet	–	–	–	0.681±0.014	0.796±0.012	0.914±0.008
CC-VLT (Proposed)	0.702±0.014	0.812±0.012	0.931±0.008	0.716±0.013	0.826±0.011	0.942±0.007

**Table 24:** Clinical reliability metrics under cross-center evaluation.

Method	CBIS-DDSM		INbreast		VinDr-Mammo	
	NPV	PPV	NPV	PPV	NPV	PPV
ResNet-50	0.841±0.011	0.782±0.014	0.857±0.010	0.798±0.013	0.862±0.010	0.804±0.012
MFAN	0.863±0.010	0.801±0.013	0.879±0.009	0.816±0.012	0.883±0.009	0.821±0.011
StethoNet	–	–	0.891±0.008	0.829±0.011	0.895±0.008	0.834±0.010
CC-VLT (Proposed)	0.902±0.008	0.835±0.011	0.917±0.007	0.852±0.010	0.921±0.007	0.858±0.009

**Table 25:** Comparison of calibration performance with post hoc calibrated baseline models.

Model	ROC–AUC	ECE ↓	Brier Score ↓	Calibration Strategy
Image-only transformer	0.901±0.007	0.084±0.005	0.192±0.006	None
Image-only + Temperature Scaling	0.901±0.007	0.051±0.004	0.171±0.005	Post hoc temperature scaling
Image-only + Isotonic Regression	0.899±0.008	0.047±0.004	0.166±0.005	Post hoc isotonic regression
BiomedCLIP adaptation	0.923±0.005	0.041±0.003	0.152±0.004	Intrinsic multimodal learning
CC-VLT (Proposed)	0.934±0.005	0.029±0.003	0.136±0.004	Intrinsic multimodal + cross-center calibration

↓ indicates that lower values represent better calibration performance.

**Table 26:** Statistical significance analysis of ROC–AUC improvements under cross-center evaluation.

Comparison	Mean ROC–AUC Gain	Paired *t*-Test *p*-Value	Wilcoxon *p*-Value
CC-VLT vs. ResNet-50	0.071±0.006	<0.001	0.003
CC-VLT vs. MFAN	0.048±0.005	<0.001	0.005
CC-VLT vs. BiomedCLIP	0.011±0.003	0.008	0.012
CC-VLT vs. CORAL-style alignment	0.011±0.004	0.011	0.018

**Table 27:** Comparative novelty analysis between the proposed CC-VLT framework and existing state-of-the-art approaches.

Method Category	Multimodal Learning	Cross-Modal Attention	Cross-Center Robustness	Structured Clinical Descriptor Integration	Fine-Grained Semantic Alignment	Mammography-Specific Optimization
CNN-based mammography models	No	No	Limited	No	No	Partial
Attention-enhanced CNNs	No	Limited visual attention	Limited	No	No	Partial
Multi-view transformer models	No	Visual attention only	Moderate	No	No	Yes
CLIP-style medical VL models	Yes	Global image–text alignment	Limited	Partial	Limited	Limited
Report-aligned radiology transformers	Yes	Cross-modal transformer	Moderate	Full reports required	Moderate	Task-dependent
Domain generalization frameworks	No	No	Yes	No	No	Partial
CC-VLT (Proposed)	Yes	Bidirectional patch–token attention	Yes	Yes	Yes	Yes

## Data Availability

The implementation of this work can be found at https://doi.org/10.5281/zenodo.20288183.
